# Visible Light–Driven Advanced Oxidation Processes to Remove Emerging Contaminants from Water and Wastewater: a Review

**DOI:** 10.1007/s11270-022-05831-2

**Published:** 2022-09-03

**Authors:** Piotr Zawadzki

**Affiliations:** Department of Water Protection, Central Mining Institute, Plac Gwarków 1, 40-166 Katowice, Poland

**Keywords:** Contaminants of emerging concern, Advanced oxidation process, Photocatalysis, Persulfate radical, Photoelectrocatalysis, Visible light

## Abstract

The scientific data review shows that advanced oxidation processes based on the hydroxyl or sulfate radicals are of great interest among the currently conventional water and wastewater treatment methods. Different advanced treatment processes such as photocatalysis, Fenton’s reagent, ozonation, and persulfate-based processes were investigated to degrade contaminants of emerging concern (CECs) such as pesticides, personal care products, pharmaceuticals, disinfectants, dyes, and estrogenic substances. This article presents a general overview of visible light–driven advanced oxidation processes for the removal of chlorfenvinphos (organophosphorus insecticide), methylene blue (azo dye), and diclofenac (non-steroidal anti-inflammatory drug). The following visible light–driven treatment methods were reviewed: photocatalysis, sulfate radical oxidation, and photoelectrocatalysis. Visible light, among other sources of energy, is a renewable energy source and an excellent substitute for ultraviolet radiation used in advanced oxidation processes. It creates a high application potential for solar-assisted advanced oxidation processes in water and wastewater technology. Despite numerous publications of advanced oxidation processes (AOPs), more extensive research is needed to investigate the mechanisms of contaminant degradation in the presence of visible light. Therefore, this paper provides an important source of information on the degradation mechanism of emerging contaminants. An important aspect in the work is the analysis of process parameters affecting the degradation process. The initial concentration of CECs, pH, reaction time, and catalyst dosage are discussed and analyzed. Based on a comprehensive survey of previous studies, opportunities for applications of AOPs are presented, highlighting the need for further efforts to address dominant barriers to knowledge acquisition.

## Introduction

The problem of contaminants of emerging concern (CECs) is an issue that is constantly being developed. CECs have been identified in groundwater and surface water, in treated municipal and industrial wastewaters, and even in drinking water (Bolong et al., 2009; Coadou et al., 2017; Montagner et al., 2019; Tröger et al., 2018). New groups of compounds have also been reported as potential substances classified as emerging contaminants: halogenated methanesulfonic acids (MSAs) such as chloro-, bromo-, or iodo-methanesulfonic acids (Zahn et al., 2016); microplastics (MPs) (Wright & Kelly, 2017); flame retardants including tetrabromobisphenol A (TBBPA) (Ballesteros-Gómez et al., 2017); compounds used in ultraviolet (UV) filters and sun creams such as ethylhexyl dimethylaminobenzoate and benzocaine (Li et al., 2017; Tsui et al., 2017); contrast agents used in computed tomography such as those containing gadolinium (Rogowska et al., 2018); pharmaceutical substances such as lidocaine (Jakab et al., 2020); and even drugs such as cocaine and its metabolites identified in pool waters (Fantuzzi et al., 2018).

The presence of contaminants of emerging concern in the environment is not normally related to their negative impact on living organisms at high doses (acute toxicity). Their low concentrations in water and wastewater (c.a. few ng/dm^3^) and long-term effects on humans and animals (chronic toxicity) should be of potential concern. The toxic effects of these substances have been confirmed by, e.g., Leusch et al. (2017) and Lempart et al. (2020).

Since these compounds are often identified in the environment and because of their negative impact on living organisms, it is justified to develop new technologies of water treatment and municipal and industrial wastewater treatments. The scientific data reviews show that one of the interesting alternatives to the conventional processes used in environmental engineering is advanced oxidation processes (AOPs). The common feature of AOPs is the physicochemical reaction between the generated hydroxyl radical (^•^OH) or sulfate radical (${\mathrm{SO}}_{4}^{\bullet -}$) and organic contaminants. AOPs are non-selective and allow the complete or partial decomposition of hazardous substances by mineralization into environmentally neutral, simple chemical compounds (Mazivila et al., 2019; Wacławek et al., 2017).

The differences between the various AOPs are the radical generation method, efficiency, and complexity. Most of them are photochemical processes, i.e., conducted in the presence of ultraviolet radiation (*λ* < 400 nm). A significant drawback is a catalytic activity, which requires using an expensive catalyst activation method with artificial light sources. The most frequently used radiation source is a lamp emitting radiation below 400 nm (UV light). This is essential for activating catalysts such as titanium(IV) oxide. Lasers, solar radiation, xenon, and sodium lamps are rarely used. When solar radiation is used, only 3–5% of this energy can be utilized, so the use of UV lamps, as energy-intensive devices, is a severe limiting factor for using these methods in the elimination of micropollutants (Ghernaout & Elboughdiri, 2019; Palit, 2014).

The scientific data also show that many works are devoted to using sulfate radicals ${\mathrm{SO}}_{4}^{\bullet -}$ (*E*^0^ = 2.5–3.1 V) for the degradation of organic contaminants (Hu et al., 2020b; Zhou et al., 2020). The generation of sulfate radicals is carried out by activation of persulfate ions (${\mathrm{S}}_{2}{\mathrm{O}}_{8}^{2-}$) by UV radiation, heat, ionizing radiation, high pH > 11.0, and transition metal ions (Criquet and Karpel Vel Leitner, 2012; Peng et al., 2017; Manz et al., 2018; Al Hakim et al., 2019). Activation with transition metal ions at low oxidation levels such as Fe^2+^, Ni^2+^, Co^2+^, and Ag^+^ is used most frequently. As a result of the reaction, the ion ${\mathrm{S}}_{2}{\mathrm{O}}_{8}^{2-}$ reacts with the electron donor from the transition metal to form a single sulfate radical (Nasseri et al., 2017).

**Fig. 1:**
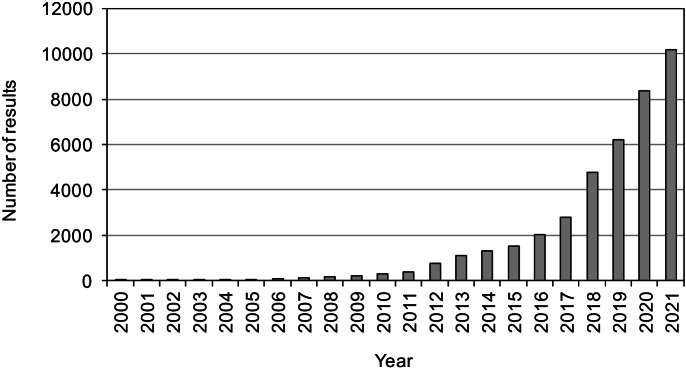
Trend of the number of publications per year search by words in the Scholar database: “visible-light driven advanced oxidation processes” from the years 2000–2021

A review of results in the Scholar database (Google Scholar Database, 2022) showed that, in recent years, there have been an increasing number of studies on the application of modifications of advanced oxidation processes, including the visible light–driven AOPs (Fig. 1). In recent years, researchers have focused on modifications of advanced oxidation processes (Cheng et al., 2019; Zawadzki et al., 2020; Zawadzki, 2020; Nguyen et al., 2020). Modifications simplify the way catalysts are activated, and increase the degree of pollutant removal efficiency with variable wastewater quality.

One example of modifications used in heterogeneous oxidation processes (in the presence of solid catalysts, e.g., titanium dioxide (TiO_2_)) is carbonaceous materials (e.g., activated carbon), acids (e.g., succinic acid, ascorbic acid), or metal and non-metal species (e.g., carbon, nitrogen, sulfur, ferrum). Activation processes of persulfates (precursors of sulfate radicals) under visible light have also attracted widespread interest (Du et al., 2020; Wang et al., 2019; Zawadzki, 2019; Zhang et al., 2020), and it is described as effective as conventional activation methods. Appropriately chosen treatment parameters and optimal modification methods can lower costs compared to classical methods.

The increasing pressure of contaminants on the environment, combined with the scarcity of water resources in the world, justifies the need to develop new and optimize already applied methods for the efficient removal of contaminants from water and wastewater. This work presents examples of methods for the advanced oxidation of micropollutants such as chlorfenvinphos, methylene blue, and diclofenac carried out in the presence of visible light.

**Fig. 2:**
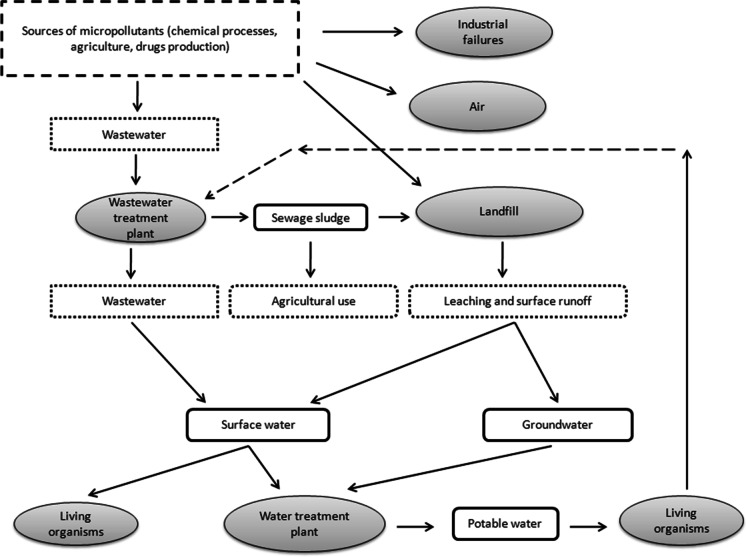
Migration of micropollutants in the environment

Micropollutants have a high susceptibility to migrate in the environment and thus bioaccumulate and migrate in the environment (Fig. 2). Micropollutants are also relatively resistant to decomposition. The emission of micropollutants to the environment is mainly due to industrial activities. It is primarily connected with thermal and chemical processes. Coking plants, power plants, waste incineration plants, and chemical plants are direct sources of micropollutants. Excessive amounts of pesticides, pharmaceuticals, and antibacterial substances are of great importance. Micropollutants are primarily identified in surface water, but their concentration is also increasing in groundwater. They are mainly transported to aquatic ecosystems with treated or poorly treated industrial and domestic wastewaters, atmospheric precipitation, and through surface runoff from agricultural land and poorly protected landfills (Dubey et al., 2021; Menger et al., 2021; Ngweme et al., 2021).

The following representatives from a group of CECs were selected as model contaminants: chlorfenvinphos, methylene blue, and diclofenac. These contaminants differ from each other in their physicochemical properties, degree of impact on living organisms, and persistence in the environment (Table 1).

**Table 1:** Toxicological and physicochemical profile of CFVP, MB, and DCF

Compound	CFVP	MB	DCF
Chemical formula	C_12_H_14_Cl_3_O_4_P	C_16_H_18_CIN_3_S	C_14_H_11_Cl_2_NO_2_
Molecular weight (g/mol)	359.6	319.85	296.1
CAS number	470–90-6	61–73-4	15,307–86-5
Form	Amber liquid	Dark green crystals or powder	Solid; crystals from ether–petroleum ether
Toxicity	*Daphnia magna*: LC_50_ (24 h) = 28.00 µg/l *Daphnia magna*: EC_50_ (24 h) = 1.2 µg/l *Gammarus fasciatus*: LC_50_ (24 h) = 27 µg/l *Gammarus fasciatus*: LC_50_ (96 h) = 9.6 µg/l	*Daphnia*: EC_50_ (48 h) = 2.26 mg/l Fish: LC_50_ (96 h) = 18.0 mg/l	*Dunaliella tertiolecta* (phytoplankton): EC_50_ (96 h) = 185.69 mg/l *Daphnia magna*: EC_50_ (48 h) = 123.30 mg/l
log *K*_OW_ (–)	3.81	5.85	4.51
log *K*_OC_ (–)	2.44	ND	3.81–4.30 ^a^
Bioconcentration factor (BCF) (–)	36.6–661.0	< 100	10
Solubility in water (at 25 °C) (mg/l)	124.0 (at 20 °C)	43,600.0	2.37
Vapor pressure at 25 °C (mmHg)	7.5 × 10^−6^	7.0 × 10^−7^	6.14 × 10^−8^
Environmental concentration (µg/l)	Surface water: 0.001–47.4 Seawater and groundwater: 0.02 Rainwater: 0.05–0.12 Wastewater (effluent): 0.05–0.14	10 × 10^3^–10 × 10^5 b^	Surface water: 4.62 × 10^−3^–0.057 Groundwater: 2.5 × 10^−3^–13.48 Drinking water: 2.5 × 10^−3^–56 ng/l Wastewater effluent: 4.5 × 10^−3^–19.0
References	Chlorfenvinphos (Safety Data Sheet 2022Zgheib et al., 2012; Campo et al., 2013; Ccanccapa et al., 2016Pérez-Lucas et al., 2018) Chlorfenvinfos (Compound Summary 2022)	Methylene blue (Material Safety Data Sheet 2022Rahman et al., 2012; Almaamary et al., 2017) Methylene blue (Compound Summary 2022)	Scheytt et al. (2005) DeLorenzo and Fleming (2008) Memmert et al. (2013) De Oliveira et al. (2016) Sathishkumar et al. (2020) Diclofenac (Compound Summary 2022)

*CFVP* chlorfenvinphos, *MB* methylene blue, *DCF* diclofenac, *ND* no data.

^a^log *K*_OC_ values based on equations by Karickhoff et al. (1979): log *K*_OC_ = 1.0 log *K* − 0.21.

^b^No data about MB concentrations in wastewater was found: the presented data refer to initial concentrations removed during laboratory experiments.

In recent years, the number of publications on the applications of AOPs based on hydroxyl or sulfate radicals has been widely studied for water, wastewater, and soil treatment (Ghernaout & Elboughdiri, 2021; Lee et al., 2020; Ma et al., 2021; Miklos et al., 2018; Zhou et al., 2019). Despite numerous publications and reviews on AOPs, there is a lack of collected and systematized information regarding visible light–driven advanced oxidation processes for the removal of emerging contaminants. Very few studies reviewed this treatment technology (Serpone et al., 2017; Tian et al., 2022; Yang et al., 2021); therefore, there is a paucity of a broad overview of its application and discussion of influencing parameters. Although the large number of publications on conventional AOPs (e.g., using UV) shows great success, due to the energy crisis and the continuously increasing electricity prices, it is important to present the potential, possibilities, and influencing parameters affecting AOPs using solar radiation as a renewable energy source. Thus, solar radiation can be applied as a free source of energy, reducing operating costs on an industrial scale. Therefore, this review article summarizes various methods of removing contaminants of emerging concern from water and wastewater by visible light–driven AOPs. An important aspect in the work is the analysis of process parameters affecting the degradation process to enhance the efficiency of the visible light–driven AOP system. Specifically, the main mechanisms involved in visible light activation are also discussed. Concluding perspectives and guidelines for future research are proposed at the end of this paper.

### Methylene Blue

Industrial wastewater contains chemical compounds (e.g., dyes, phenols, pesticides, heavy metals), which are by-products of the technological processes, e.g., in the textile, chemical, food, and tanning industries. These industries generate the most significant industrial wastewater containing synthetic dyes (Khamparia & Jaspal, 2018). The composition of colored industrial effluents is chemically diverse. The synthetic dyes commonly found in this type of wastewater can be divided into azo dyes, reactive dyes, triphenylmethane dyes, heterocyclic dyes, and polymeric dyes (Guadie et al., 2017).

One example of a compound belonging to the group of azo dyes is methylene blue (MB). Azo dyes contain the azo functional group R-N = N-R′, in which R and R′ can be substituted with alkyl or aryl groups. Azo dyes are one of the leading organic compounds identified in colored industrial effluents and can account for up to 70% of total dye production (Oveisi et al., 2019). Azo dyes trigger histamine, which can cause, for example, urticaria and can aggravate asthma symptoms and cause uterine contractions in pregnant women, causing miscarriage. MB is a cationic thiazine dye containing a six-membered heterocyclic ring with sulfur and a nitrogen atom (phenothiazine ring). Methylene blue is widely used in the textile, paper, cosmetics, plastics, and food industries (Zawadzki, 2019). Recent literature also indicates the potential use of MB in treating COVID-19, a disease caused by the SARS-CoV-2 virus (Gendrot et al., 2020; Scigliano & Scigliano, 2021).

For organic substances with a log *K*_OW_ value less than 4.5, the affinity for the lipids of the organism is assumed to be insufficient to exceed the bioaccumulation criterion (bioconcentration factor (BCF) = 2000). The BCF is the ratio of the concentration of a substance in an organism and water, depending on the organism and the conditions. Methylene blue exceeds this factor (log *K*_OW_ = 5.85). Chemicals with high log *K*_OW_ values (> 4.5) are of more significant concern as they have the potential to bioconcentrate in living organisms. However, MB is not expected to bioaccumulate significantly as the estimated BCF is below 100.

Due to the potential danger to humans and the high resistance to biodegradation, there is a need to develop technologies to eliminate methylene blue from water and wastewater. Removal of dyes by conventional processes, including activated sludge, does not bring the expected results. Due to their low biodegradability, almost 90% of the dyes present in wastewater is not removed by conventional treatment processes. Therefore, the degradation of dyes from wastewater has attracted considerable interest from researchers worldwide (Deng and Zhao, 2015). In recent years, interesting, advanced oxidation processes driven by visible light have been developed to remove methylene blue from colored wastewater.

### Chlorfenvinphos

For many years, interest in pesticides has focused on four basic properties: selective toxicity, persistence in the environment, bioaccumulation, and mobility. Persistence in the environment is probably the most decisive factor when considering the extent of their use. Persistence is often expressed in terms of half-life. Pesticide degradation can occur through biological processes and chemical and photochemical reactions. A pesticide losing its characteristic activity does not necessarily mean that it has become a harmless substance. Chemical reactions often result in more toxic compounds than the original compounds (Mahdy & El-Maghraby, 2010; Laws 2013; Ravoet et al., 2015).

Chlorfenvinphos (CFVP) is one of the most important members of the organophosphorus insecticide family. Technical chlorfenvinphos, consisting of E and Z isomers, contains about 80–90% of this compound. CFVP is a low-mammalian toxicity insecticide. It is used against pests destroying crops of potatoes, rice, carrots, oilseeds, and maize. Organophosphate insecticides are phosphoric acid derivatives in which the hydroxyl group (–OH) has been replaced by –OR groups derived from alcohols. Organophosphate pesticides inhibit the activity of acetylcholinesterase, one of the essential enzymes for the peripheral and central nervous systems. Chlorfenvinphos can cause structural and functional changes in the liver (Lutz et al., 2006; Sismeiro-Vivas et al., 2007; Sosnowska et al., 2013).

Chlorfenvinphos has a moderate bioconcentration potential as indicated by a log *K*_OW_ value of 3.81. The degree of bioconcentration of CFVP ranges from BCF = 36.6 to 661.0. The organic carbon/soil partition coefficient (log *K*_OC_) value is approximately 2.44. The log *K*_OC_ value shows a moderate susceptibility to adsorb in bottom sediments and suspended matter, and therefore, the transport of the compound to the solid phase is to be expected. CFVP hydrolyzes slowly in slightly alkaline, acidic, and neutral conditions. The half-life (*t*_1/2_) at pH 3 to 6 is between 170 and 200 days (*T* = 20–30 °C). Chlorfenvinphos is more resistant to decomposition in biologically active waters, with half-lives ranging from 1 to 25 days. It only decomposes thermally at high temperatures (*T* > 150 °C). Despite its ban in Europe, it is identified in water samples worldwide (Serrano et al., 1997; Wu et al., 2011; Sire and Amouroux, 2016; Ccanccapa et al., 2016; Koranteng et al., 2018; Pérez-Lucas et al., 2018).

The number of publications on the application of advanced processes to eliminate chlorfenvinphos is not significant. The number of results in the Scholar database in 2000–2021 containing the phrase “advanced oxidation process for chlorfenvinphos removal” is 1050, while that containing the words “visible-light-driven advanced oxidation process for chlorfenvinphos removal” is 154. Compared to atrazine, an herbicide from the triazine group (17,500 in 2000–2021 for the phrase “advanced oxidation process for atrazine removal” and 5170 for the phrase “visible-light-driven advanced oxidation process for atrazine removal”), diuron, a phytotoxic herbicide from the group of total herbicides (12,400 for “advanced oxidation process for diuron removal” and 1220 for “visible-light-driven advanced oxidation process for diuron removal”), has a relatively low number. Therefore, there is a gap between the removal processes for individual organophosphorus pesticides investigated so far. This is important because atrazine, diuron, and chlorfenvinphos are on the list of priority substances for water policy (Directive, 2013).

### *Diclofenac*

Among the analyzed CECs, there is a group of pharmaceutical substances. The largest pharmaceutical substances come from hospitals, households, veterinary centers, and livestock farms. Municipal wastewater discharge is considered the dominant source of pharmaceuticals, while discharges from manufacturing plants, hospitals, and farms are locally significant (Wöhler et al., 2020). Pharmaceuticals are designed to perform a precise function in the human body. Significant fractions of pharmaceutical substances are generally excreted, mainly through urine (Barreto et al., 2021). Pharmaceutical products for use in both humans and animals are developing together with the global population increase and healthcare. The number of pharmaceuticals discharged into the environment is an increasingly severe problem. More than 3500 pharmaceutical substances have been identified in surface water and treated wastewater, excluding metabolites and other transformation products (Aissaoui et al., 2017).

Diclofenac (DCF) is a non-steroidal anti-inflammatory drug (NSAID) (Sathishkumar et al., 2020). Anti-inflammatory painkillers are among the most popular drugs available, mostly over-the-counter. The most commonly purchased painkillers include ibuprofen, paracetamol, naproxen, diclofenac, carbamazepine, and salicylic acid. Diclofenac is used in both humans and livestock. Worldwide annual consumption of diclofenac is estimated to be around 1000 mg (Moreira et al., 2018; Tomul et al., 2019).

The widespread use of pharmaceuticals results in the almost continuous emission of these compounds and their metabolites into the environment. The increased importance of pharmaceutical substances has prompted several actions to limit or monitor these compounds. For example, by Commission Implementing Decision (EU) No. 2015/495 of 20 March 2015 establishing a watch list of substances for monitoring purposes, diclofenac was included in the first watch list. According to the current Commission Implementing Decision (EU) No. 2020/1161 of 4 August 2020 establishing a watch list of substances for monitoring purposes, as many as four compounds from the group of pharmaceuticals have been included: amoxicillin (a semi-synthetic β-lactam antibiotic with bactericidal activity), ciprofloxacin (a second-generation quinolone chemotherapeutic with bactericidal activity), sulfamethoxazole (a bacteriostatic antibiotic), trimethoprim (a chemotherapeutic agent), venlafaxine, and *O*-desmethylvenlafaxine (a multifunctional organic chemical compound used as an antidepressant) (Commission Implementing Decision (EU) 2015/495; Commission Implementing Decision (EU) 2020/1161). Furthermore, Font et al. (2019) developed a model to predict the current and future dilution of pharmaceuticals in freshwater ecosystems such as rivers and lakes. Their model was applied to diclofenac, a commonly used anti-inflammatory drug to reduce pain.

Approximately 65% of the diclofenac dose is excreted in urine and 35% in bile as conjugates of unchanged diclofenac and its metabolites (Voltaren‒Prescribing Information 2022). Diclofenac tends to adsorb to the organic matter in soil or sediments due to their low affinity for water (log *K*_OW_ = 4.51 and log *K*_OC_ = 3.81–4.30). The bioconcentration degree of DCF is 10 (diclofenac is not expected to bioaccumulate significantly).

Conventional treatment processes have DCF removal efficiencies ranging from a few % to 93% (Lonappan et al., 2016; Verlicchi et al., 2012). Zhang et al. (2008) reported diclofenac removal efficiencies by wastewater treatment plants ranging from 0 to 80%, mainly in the 21–40% range. Zorita et al. (2009) showed that the DCF reduction factor could also be harmful, which is attributed to de-conjugation or hydrolysis of pharmaceutical metabolites, reformation of the parent molecule, or pharmaceutical desorption from colloids and suspension (sewage sludge). Therefore, DCF persists in the aquatic environment and is detected in raw wastewater, treated wastewater, surface water, and even drinking water (Loos et al., 2017; Sharma et al., 2019).

## Visible Light–Driven Advanced Oxidation Processes

There are currently many technologies for the removal of emerging contaminants. These include chemical precipitation, flotation, adsorption on activated carbon, wet air oxidation, supercritical water oxidation, Fenton’s reagent, hydrogen peroxide treatment, ultrasonic oxidation, ozonation, membrane processes (microfiltration, ultrafiltration, reverse osmosis, electrodialysis), and biological processes, as well as combined processes such as membrane bioreactors (biological processes and membrane processes) or biological activated carbons (Dhaka et al., 2019; Gogoi et al., 2018; Rodriguez-Narvaez et al., 2017). However, the above methods have some disadvantages. For example, coagulation generates large amounts of sludge formation and additional equipment for sedimentation and filtration of the resulting sludge is required. Sorption processes require optimal adsorbents with a high affinity for the contaminants. On the other hand, membrane processes require pre-treatment of the wastewater to eliminate substances that limit the life of the membranes. Wet air oxidation uses air as an oxidant mixed with the contaminated medium and then passed through a catalyst with increased temperature and pressure (high-temperature process and high electricity consumption are limitations of this process). In contrast, microorganisms in biological methods are sensitive to toxic substances, and pre-treatment before biological treatment is required. Most of the mentioned methods do not degrade the contaminants but only transfer them to another phase, so CECs are still present in the environment (Tungler et al., 2015; De Gisi et al., 2016; Obotey Ezugbe & Rathilal, 2020).

In recent years, the interest of researchers has focused on the development of AOPs that, under appropriately chosen conditions (e.g., reaction time, oxidant dose, reactor volume), allow the degradation of almost 100% of the contaminants and minimize the risk of generating oxidation by-products. The use of energy-consuming UV lamps (Zou et al., 2020), the recombination of hole-electron pairs (photocatalysts) (Sharma et al., 2021), the influence of interfering ions (Ahmed et al., 2021), the result of suspended matter (slurry), the formation of post-process residues (nanoparticles of photocatalysts, negatively affecting ecosystems and human health by entering, for example drinking water sources) (Zhang et al., 2017), and the use of energy- and cost-intensive activation methods (high temperature, high pH, chemical reactants, e.g., Fe^2+^ for radical generation processes ${\mathrm{SO}}_{4}^{\bullet -}$) (Hu et al., 2020a, 2020b; Zawadzki, 2019; Zrinyi & Pham, 2017) are significant limitations of AOPs. Therefore, new and efficient, technologically and economically effective processes are sought in environmental engineering to achieve simultaneously high removal results of CECs. The analysis of literature data has shown that three basic types of visible light–driven AOPs are used for CFVP, DCF, and MB elimination processes: photocatalysis, radical sulfate oxidation, and electrochemical processes. A summary of the identified visible light–driven processes is presented in Table 2.

**Table 2:** The experimental conditions and removal efficiency of visible light–driven AOPs for CFVP, MB, and DCF degradation

CEC	Process	Removal efficiency (%)	Details	References
Chlorfenvinphos	Visible light–driven photoelectrochemical degradation in the presence of WO_3_ nanosheets/nanorods	95	Thermal treatment (annealing) of nanostructured electrodes = 600 °C; concentration *C*_0[CFVP]_ = 20 mg/l; process time = 360 min; pH = 1; Temp. = 20 °C; the bias potential (vs. SCE) = + 1 V; type of lamp = Xe lamp; lamp power = 1000 W	Fernández-Domene et al. (2019)
Chlorfenvinphos	Photocatalysis in the presence of pyruvic acid (PA)-doped TiO_2_ (TiO_2_/PA)	85	Concentration *C*_0[CFVP]_ = 1.0 mg/l; adsorption time = 20 min; process time = 60 min; catalyst dosage = 50 mg/l; pH = 3; type of lamp = tungsten; lamp power = 10 W	Zawadzki (2020)
Chlorfenvinphos	Photodegradation by using WO_3_ nanostructures	95	Thermal treatment (annealing) of nanostructured electrodes = 600 °C; anodization in electrolyte: 1.5 M CH_4_O_3_S; 0.05 M H_2_O_2_; concentration *C*_0[CFVP]_ = ND; process time = 1440 min; the bias potential (vs. SCE) = + 1 V; Temp. = room temperature; type of lamp = Xe lamp; lamp power = 500 W	Roselló-Márquez et al. (2021)
Chlorfenvinphos	Visible (Vis) light activation of persulfate (PS) by glucose (PS/Vis/Glu)	81	Concentration *C*_0[CFVP]_ = 1 mg/l; glucose dosage = 100 mM; PS dosage = 20 mM; process time = 20 min; pH = 6; type of lamp = tungsten; lamp power = 10 W	Zawadzki (2021b)
Methylene blue	Photocatalysis in the presence of copper phthalocyanine-sensitized TiO_2_ nanopowders (CuPc/TiO_2_)	70	Concentration *C*_0[MB]_ ≈ 192 mg/l; catalyst dosage ≈ 333.33 mg/l; concentration of Cu = 4.7 wt%; adsorption time = 30 min; process time = 150 min; Temp. = room temperature; type of lamp = xenon; lamp power = 150 W	Cabir et al. (2017)
Methylene blue	Photocatalysis in the presence of nanostructured Fe/FeS powder	96	Concentration *C*_0[MB]_ = 5 mg/l; catalyst dosage = 1000 mg/l; adsorption time = 30 min; process time = 200 min; pH = 11; type of lamp = ND; lamp power = 400 W	Esmaili et al. (2018)
Methylene blue	Photocatalysis in the presence of CuS-CdS nanocomposite	99.97	Concentration *C*_0[MB]_ = 10 mg/l; catalyst dosage = 200 mg/l; adsorption time = ND; process time = 10 min; type of lamp = tungsten; lamp power = 250 W	Mahanthappa et al. (2019)
Methylene blue	Photocatalysis in the presence of ZnO-supported Au/Pd bimetallic nanocomposites	97	Concentration of Au = 10 wt%; concentration of Pd = 5 wt%; concentration *C*_0[MB]_ = 16 mg/l; catalyst dosage = 5 mg/l; adsorption time = 30 min; process time = 180 min; Temp. = room temperature; type of lamp = xenon; lamp power = 200 W	Lee et al. (2019)
Methylene blue	Photocatalysis in the presence of Fe_2_O_3_/graphene/CuO (FGC) nanocomposite	94	Concentration *C*_0[MB]_ = 20 mg/l; catalyst dosage = 500 mg/l; adsorption time = 120 min; process time = 40 min; Temp. = 25 °C; type of lamp = ND; lamp power = 100 W	Nuengmatcha et al. (2019)
Methylene blue	Photocatalysis in the presence of Gd-doped ZnO nanoparticles	93	Concentration of Gd = 3%; concentration *C*_0[MB]_ = 10 mg/l; catalyst dosage ≈ 360 mg/l; adsorption time = 30 min; process time = 90 min; type of lamp = LED; lamp power = 40 W	Selvaraj et al. (2019)
Methylene blue	Photocatalysis in the presence of magnetic TiO_2_/NiFe_2_O_4_/reduced graphene oxide nanocomposite	71	Concentration of graphene = 120 mg; concentration *C*_0[MB]_ = 10 mg/l; catalyst dosage = 100 mg/l; H_2_O_2_ dosage = 725 mg/l; adsorption time = 60 min; process time = 90 min; pH = 11; type of lamp = halogen; lamp power = 500 W	Ziarati Saravani et al. (2019)
Methylene blue	Photocatalysis in the presence of CdS/SnO_2_ nanoparticles	80	Concentration of CdS = 5 wt%; concentration *C*_0[MB]_ = 6.4 mg/l; catalyst dosage = 1000 mg/l; adsorption time = 30 min; process time = 180 min; Temp. = 30 − 35 °C; type of lamp = mercury; lamp power = 16 W	El-Katori et al. (2020)
Methylene blue	Photocatalysis in the presence of molybdenum disulfide composed by LDH composite (MoS_2_/LDH)	95	Ratio of molybdate to thiourea = 1:5; concentration *C*_0[MB]_ = 20 mg/l; catalyst dosage = 200 mg/l; adsorption time = 30 min; process time = 180 min; pH = 4; type of lamp = xenon; lamp power = 300 W	Chen et al. (2020)
Methylene blue	Photocatalysis in the presence of CdS-NiFe_2_O_4_/reduced graphene oxide photocatalyst	92	Concentration *C*_0[MB]_ = 10 mg/l; catalyst dosage = 400 mg/l; adsorption time = 60 min; process time = 120 min; pH = 7; Temp. = 25 °C ± 2 °C; type of lamp = mercury; lamp power = 250 W	Bagherzadeh et al. (2018)
Methylene blue	Degradation by sodium persulfate activated by glucose (PS/G/Vis)	84	Concentration *C*_0[MB]_ = 2 mg/l; process time = 90 min; glucose dosage = 100 mM; PS dosage = 0.065 mM; pH = 12; Temp. = room temperature; type of lamp = tungsten; lamp power = 10 W	Zawadzki (2019)
Methylene blue	Degradation by peroxymonosulfate (PMS) and BiVO_4_	99	Concentration *C*_0[MB]_ = 5 mg/l; BiVO_4_ dosage = 200 mg/l; PMS dosage = 1 mM; adsorption time = 10 min; process time = 90 min; pH = 6; type of lamp = LED; lamp power = 30 W	Tang (2020)
Methylene blue	PS activated by TiO_2_/FeOCl	100	Concentration of FeOCl = 20 wt%; concentration *C*_0[MB]_ = 3.2 mg/l; catalyst dosage = 400 mg/l; PS dosage = 1.48 mM; adsorption time = 60 min; process time = 90 min; Temp. = 25 °C; type of lamp = LED; lamp power = 50 W	Sabri et al. (2020)
Methylene blue	PS activated by Ag/Mn_3_O_4_ (Ag/Mn_3_O_4_-0.5)	82	Ag:Mn_3_O_4_ ratio = 1:0.5; concentration *C*_0[MB]_ = 40 mg/l; catalyst dosage = 500 mg/l; process time = 90 min; PS dosage = 12 mM; pH = 7; Temp. = room temperature; type of lamp = xenon; lamp power = 40 W	Rizal et al. (2021)
Methylene blue	PS activated by Ag/Mn_3_O_4_/graphene composites (Ag/Mn_3_O_4_-5G)	100	Ag:Mn_3_O_4_ ratio = 1:0.5; graphene composites concentration = 5 wt%; concentration *C*_0[MB]_ = 40 mg/l; catalyst dosage = 500 mg/l; process time = 60 min; PS dosage = 12 mM; pH = 7; Temp. = room temperature; type of lamp = xenon; lamp power = 40 W	
Methylene blue	PMS activated by surface-tailored carbon quantum dots (CQDs)	90.1	Concentration *C*_0[MB]_ = 20 mg/l; PMS dosage = 246 mg/l; catalyst dosage = 4000 mg/l; adsorption time = 30 min; process time = 60 min; Temp. = 25 °C; type of lamp = LED; lamp power = 40 W	Han et al. (2020a)
Methylene blue	Photoelectrocatalysis in the presence of cadmium sulfide–sensitized titanium dioxide film	88	Number of CdS layer = 6; concentration *C*_0[MB]_ = 5 mg/l; adsorption time = 60 min; effective area of photoelectrode = 2 cm^2^; the bias potential (vs. SCE) = + 4 V; process time = 180 min; type of lamp = xenon; lamp power = 300 W	Wu et al. (2019)
Methylene blue	Photoelectrocatalysis in the presence of F-doped TiO_2_ photoelectrode	92	Concentration of F = 15 wt%; concentration *C*_0[MB]_ = 10 mg/l; adsorption time = 30 min; effective area of photoelectrode = 50 cm^2^; the bias potential (vs. SCE) = + 1.4 V; pH = 6.89; process time = 240 min; type of lamp = metal halide; lamp power = 450 W	Liu et al. (2017a)
Methylene blue	Photoelectrocatalysis in the presence of ZnO-coated nanoporous silicon by atomic layer deposition	88	Concentration *C*_0[MB]_ = 20 mg/l; adsorption time = 120 min; effective area of photoelectrode = 16 cm^2^; the bias potential (vs. SCE) = + 6 V; process time = 105 min; type of lamp = xenon; lamp power = 300 W	Sampath et al. (2016)
Methylene blue	Photoelectrocatalysis in the presence of TiO_2_-decorated CuCr_2_O_4_ (CCO) nanocomposite	97.28	Concentration *C*_0[MB]_ = 10 mg/l; CCO dosage = 400 mg; adsorption time = 30 min; effective area of photoelectrode = 0.12 cm^2^; catalyst dosage ≈ 833 mg/l; H_2_O_2_ dosage = 4 mM; the bias potential (vs. SCE) = + 0.71 V; process time = 15 min; *T* = room temperature; type of lamp = LED; lamp power = 50 W	Ghorai et al. (2021)
Methylene blue	Photoelectrocatalysis in the presence of Cu_2_O photocathode in conjunction with a WO_3_/BiVO_4_	97	Concentration *C*_0[MB]_ = 5 mg/l; the bias potential (vs. SCE) = + 0.4 V; process time = 180 min; type of lamp = tungsten; lamp power = 60 W	Thongthep et al. (2021)
Methylene blue	Photoelectrocatalysis in the presence of CdMoO_4_/g-C_3_N_4_ nanocomposite (CMO/CN)	95	CMO:CN ratio = 10 wt%; concentration *C*_0[MB]_ = 10 mg/l; adsorption time = 30 min; effective area of photoelectrode = 0.071 cm^2^; process time = 150 min; type of lamp = xenon; lamp power = 35 W	Gandamalla et al. (2021)
Methylene blue	Photoelectrocatalysis in the presence of FTO/WO_3_/BiVO_4_	94	Concentration *C*_0[MB]_ = 5 mg/l; the bias potential (vs. SCE) = + 2 V; process time = 180 min; type of lamp = ND; lamp power = 20 W	Nareejun and Ponchio (2020)
Methylene blue	Photoelectrocatalysis in the presence of In_2_O_3_-ZnO nanocomposites	95	In:Zn ratio = 0.05:1 (5%); concentration *C*_0[MB]_ = 20 mg/l; adsorption time = 60 min; effective area of photoelectrode = 4 cm^2^; the bias potential (vs. SCE) = + 0.2 V; process time = 60 min; type of lamp = xenon; lamp power = ND	Zhao et al. (2019)
Diclofenac	Photocatalysis in the presence of tungsten trioxide–doped TiO_2_ (TiO_2_-WO_3_)	91	Concentration *C*_0[DCF]_ = 25 mg/l; pH = 5; catalyst dosage = 600 mg/l; adsorption time = 30 min; process time = 240 min; type of lamp = metal halide; lamp power = 400 W	Mugunthan et al. (2018)
Diclofenac	Photocatalysis in the presence of vanadium oxide/boron-co-doped graphitic carbon nitride (V_2_O_5_-BCN)	80–100	Concentration *C*_0[DCF]_ = 5–50 mg/l; pH = 5–9; catalyst dosage = 500–2000 mg/l; process time = 120 min; type of lamp = monochromatic blue; lamp power = 8 W	Oliveros et al. (2021)
Diclofenac	Photocatalysis in the presence of bismuth oxychloride/graphene oxide (BiOCl-GO) composite	95	Concentration *C*_0[DCF]_ = 5 mg/l; pH = 6; catalyst dosage = 1000 mg/l; process time = 120 min; type of lamp = mercury; lamp power = 96 W (12 × 8 W)	Rashid et al. (2020)
Diclofenac	Photocatalysis in the presence of tungsten trioxide–doped ZnO (ZnO-WO_3_)	90	Concentration *C*_0[DCF]_ = 15 mg/l; pH = 6; catalyst dosage = 800 mg/l; adsorption time = 30 min; process time = 270 min; type of lamp = metal halide; lamp power = 400 W	Mugunthan et al. (2019)
Diclofenac	Photocatalysis in the presence of Ti-doped BiOI microspheres (TB450)	99.2	Concentration *C*_0[DCF]_ = 10 mg/l; pH = 5; catalyst dosage = 250 mg/l; process time = 90 min; type of lamp = ND; lamp power = ND	Liu et al. (2019)
Diclofenac	Photocatalysis in the presence of cobalt(II) and cobalt(III) oxide and tungsten(VI) oxide composites (Co_3_O_4_/WO_3_)	98.7	Concentration *C*_0[DCF]_ = 10 mg/l; pH = 6.8; catalyst dosage = 30 mg/l; adsorption time = 30 min; process time = 180 min; type of lamp = mercury; lamp power = 80 W	Malefane, Feleni, & Kuvarega (2019)
Diclofenac	Photocatalysis in the presence of CQD-modified BiOCOOH photocatalysts (CQDs/BiOCOOH)	100	Concentration *C*_0[DCF]_ = 4 mg/l; pH = 7; catalyst dosage = 600 mg/l; process time = 60 min; type of lamp = xenon; lamp power = 350 W	Chen et al. (2018)
Diclofenac	Photoelectrocatalysis in the presence of persulfate activated by Cu cathode	86.3	Concentration *C*_0[DCF]_ = 10 mg/l; pH = 5.62; catalyst dosage = 10 mM; the bias potential (vs. SCE) = + 1.5 V; process time = 120 min; type of lamp = xenon; lamp power = 300 W	Liu et al. (2017b)
Diclofenac	Degradation by peroxymonosulfate activated by Co_3_O_4_-modified g-C_3_N_4_ (Co_3_O_4_-g-C_3_N_4_)	100	Concentration *C*_0[DCF]_ = 10 mg/l; pH = 6.7; catalyst dosage = 500 mg/l; process time = 30 min; type of lamp = xenon; lamp power = 50 W	Shao et al. (2017)
Diclofenac	Degradation by PMS activated by BiFeO_3_ microspheres (BFO)	82	Concentration *C*_0[DCF]_ = 0.025 mM; pH = 7; PMS dosage = 0.5 mM; BFO dosage = 500 mg/l; adsorption time = 60 min; process time = 60 min; type of lamp = LED; lamp power = ND	Han et al. (2020b)
Diclofenac	Visible light–driven photoelectrocatalytic degradation by N, S-TiO_2_/TiO_2_ NT photoelectrode	73.3	Concentration *C*_0[DCF]_ = 5 mg/l; adsorption time = 120 min; effective area of photoelectrode = 4 cm^2^; the bias potential (vs. SCE) = + 0.4 V; process time = 720 min; type of lamp = xenon; lamp power = 35 W	Cheng et al. (2015)
Diclofenac	Photoelectrocatalytic degradation at g-C_3_N_4_/BiVO_4_ composite	32	Concentration *C*_0[DCF]_ = 10 mg/l; effective area of photoelectrode = 6 cm^2^; the bias potential (vs. SCE) = + 1 V; pH = 6.52; process time = 180 min; type of lamp = xenon; lamp power = 300 W	Sun et al. (2018)
	H_2_O_2_-assisted photoelectrocatalytic degradation at g-C_3_N_4_/BiVO_4_ composite	93.4	Concentration *C*_0[DCF]_ = 10 mg/l; effective area of photoelectrode = 6 cm^2^; the bias potential (vs. SCE) = + 1 V; pH = 3.17; process time = 180 min; type of lamp = xenon; lamp power = 300 W; H_2_O_2_ dosage = 10 mM	

### Visible Light–Driven Photocatalysis

IUPAC defines photocatalysis as the initiation of a reaction or a change in its rate under the influence of solar (Vis), UV, and infrared (IR) radiation in the presence of a photocatalyst (semiconductor), which, by absorbing the radiation, participates in the transformation of the reaction substrates (Braslavsky, 2007). Photocatalysis is carried out in the presence of metal oxides, including TiO_2_, zinc oxide (ZnO), WO_2_, CeO_2_, and Fe_2_O_3_, or sulfides CdS and ZnS. Photochemical processes are most often carried out in TiO_2_. The advantage of TiO_2_ is its chemical and biological stability. Titanium(IV) oxide is non-toxic and practically insoluble. From the economic point of view, it is relatively cheap and easy to produce. In a photocatalytic process carried out in the presence of TiO_2_, it is necessary to provide radiation of an appropriate wavelength, at an energy amount higher than that of bandgap energy. The minimum energy required for its activation is equal to the energy of the excited band and is *E*_g_ = 3.02 V for rutile form and *E*_g_ = 3.2 V for anatase form. The excitation of a semiconductor causes the transfer of an electron from the valence band (VB) to the conduction band (CB). A so-called electron–hole is produced, which corresponds to the formation of redox potential on the surface of the photocatalyst molecule. Titanium dioxide can be activated with light energy with a wavelength of *λ* < 400 nm. This is only a fraction of sunlight (< 5%), so it is necessary to provide expensive lamps that emit ultraviolet radiation in the range *λ* = 300–388 nm. Among the main disadvantages of the process of photocatalytic oxidation of contaminants, the following can be mentioned: the decomposition time of contaminants, the use of energy-consuming UV lamps, the presence of substances (salts) that reduce the efficiency of contaminant removal, the nanoparticle nature of TiO_2_, and therefore, the problematic isolation from aqueous solutions, as well as the pH dependence of photodegradation process (Ameta et al., 2018; Xing et al., 2016; Zhang et al., 2018).

For the practical application of heterogeneous processes involving semiconductors, it is vital to increase the efficiency of the photocatalysis process in visible light and to immobilize titanium dioxide nanoparticles on larger surfaces. Therefore, many works are devoted to TiO_2_ modification. Currently, green photocatalysts capable of absorbing radiation in the visible light range (*λ* > 400 nm) are of great interest. Over the last years, authors of many works have attempted to produce photocatalysts active in visible light or develop methods and/or materials for semiconductor modification. This issue has been extensively discussed in the works of Parnicka et al. (2017), Liao et al. (2018), D’Amato et al. (2018), Farhadian et al. (2019), Qi et al. (2019), and Zawadzki (2020). In brief, various types of metal or non-metal dopants (e.g., carbon, silver, gold, neodymium), activated carbon (granular or powdered), graphene oxide or carbon nanotubes or biopolymers (e.g., chitosan), and organic acids (e.g., ascorbic acid, succinic acid, pyruvic acid) are used to modify semiconductors.

**Fig. 3:**
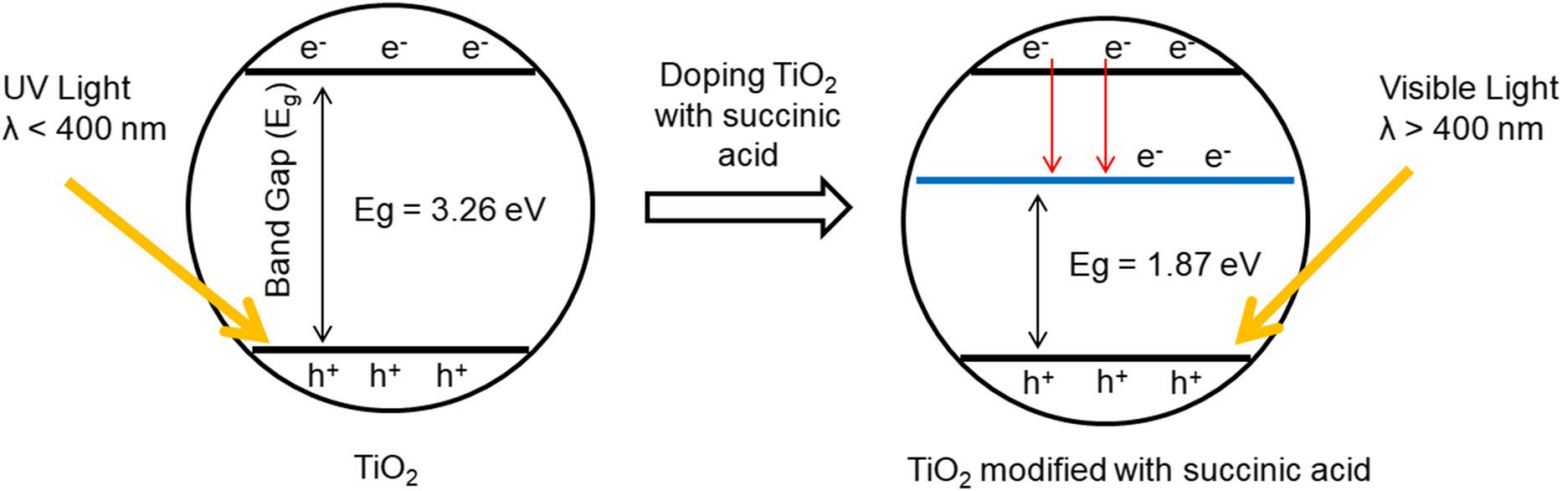
Charge transfer process in TiO_2_ modified with succinic acid under the irradiation of ultraviolet (UV) light and visible (Vis) light

The modifications are changing the structure of photocatalysts, which increases the photostability of semiconductors, and thus, their activity in visible light and better adsorption properties are observed. The adsorption of the micropollutants on the catalyst surface is the key to successful photocatalysis. Due to the nanoparticle nature of titanium dioxide and its difficult isolation from water, it has been found beneficial to modify the TiO_2_ by high porous carbon. The dopants make it possible to broaden the absorption of visible light by introducing additional energy states, inhibiting the transformation of anatase to rutile, and intensifying the conductivity of the catalysts (Fig. 3).

CFVP removal processes during visible light–driven photocatalysis have not been carried out by many studies. The use of modified titanium(IV) oxide to degrade CFVP has been studied by Zawadzki (2020). Approximately 85% degradation of CFVP (initial concentration *C*_0[CFVP]_ = 1 mg/l) in the presence of TiO_2_ modified with pyruvic acid in a 90:10 ratio (TiO_2_:pyruvic acid (PA), 90:10) was achieved. The decomposition of chlorfenvinphos was most effective under the following conditions: catalyst dose = 50 mg/l, adsorption time = 20 min, photocatalysis time = 60 min, and pH of standard solution = 3. The visible light source was a 10-W tungsten lamp. The modified titanium(IV) oxide showed activity in visible light with activation energy (*E*_g_) = 1.5 eV. The study showed that the visible light–activated TiO_2_;PA (90:10) can be used several times in the photocatalytic process. After 5 cycles, the decomposition of CFVP decreased by 12% in the presence of modified TiO_2_. Based on the study, it can be concluded that modification of TiO_2_ with organic acids can reduce the recombination of hole-electron pairs (acids are electron acceptors), similarly stated by Li Puma et al. (2008).

Residual organic pollutants may be adsorbed on the catalyst surface, successively reducing the number of active sites of the catalysts, resulting in lower catalyst performance. Modification of TiO_2_ with tungsten(VI) oxide (WO_3_) maintained a high degradation efficiency (c.a. 80%) of diclofenac in 4 reaction cycles (Mugunthan et al., 2018). The TiO_2_-WO_3_ catalyst activated under visible light allowed to obtain 91% degradation of DCF within 4 h. Some phenomena such as the recombination of hole-electron pairs, blocking of TiO_2_ active sites, or generation of reaction by-products can be reduced or eliminated due to the catalyst modifications.

Ahmed et al. (2021) reported that the advanced oxidation process can be affected by interfering ions. Such ions include, for example, Cl^−^, NO_2_^−^, NO_3_^−^, PO_4_^3−^, HCO_3_^−^, or CO_3_^2−^. Ions can inhibit the degradation process by scavenging free radicals, affecting radiation absorption, or reacting with oxidative radicals to form less reactive forms (Farner Budarz et al., 2017). The influence of interfering ions is important in AOP, also during DCF degradation, so Oliveros et al. (2021) investigated the effect of chlorides, nitrates, sulfates, and phosphates on the photocatalytic degradation of diclofenac in the presence of vanadium pentoxide (V_2_O_5_)-boron-doped graphitic carbon nitride (BCN) catalyst. The V_2_O_5_-BCN catalyst was prepared by combining V_2_O_5_ with BCN. High concentrations of anionic compounds decrease the reaction kinetics. When the concentration of negatively charged electrolytes is increased, the efficiency of DCF degradation decreases. This phenomenon is related to the competition of anionic compounds for catalytic sites and/or their reaction with oxidative radicals. A similar phenomenon was observed by Rehman et al. (2021).

In general, DCF removal efficiency depends on the following parameters: initial DCF concentration, pH of the solution, temperature, catalyst dosage, and catalyst type.

Mainly xenon, mercury, halogen, or monochromatic blue lamps ranging from a few W to 400 W are used for DCF degradation (Chen et al., 2018; Mugunthan et al., 2019; Oliveros et al., 2021; Shao et al., 2017). The time required to reach adsorption equilibrium is usually provided before photocatalysis. This time ranges from 20 to 30 min. To obtain a DCF removal above 90%, typically 30 to 270 min is needed, depending on the photocatalysis configuration.

In the process studied by Oliveros et al. (2021), the removal efficiency of diclofenac ranged from about 80% to almost 100% after 120 min of reaction. The removal rate of DCF increased with increasing catalyst dose and pH, while it decreased with increasing initial pharmaceutical concentration. Similar results were obtained by Rashid et al. (2020).

An important parameter determining the oxidation reactions occurring on the surface of photocatalysts is the pH of the solution. This parameter is related to the value of the semiconductor isoelectric point (pH_pzc_), corresponding to the pH value for which the total charge of the photocatalyst particle is zero. For TiO_2_ particles, the pH_pzc_ ranges from 6.0 to 6.5 (Kosmulski, 2011). With the change in pH, the solubility of the substance also changes. The dissociation constant of diclofenac (p*K*_a_ ≈ 4) determines its solubility: below p*K*_a_, diclofenac is insoluble, and above p*K*_a_, the DCF is negatively charged. Changing the pH also alters the electrical charge of the substances removed, resulting in a change in their ability to adsorb on the catalyst surface and the efficiency of photodegradation. Modifications of TiO_2_ can positively influence the decomposition of contaminants. For example, the amphoteric properties of titanium(IV) oxide can be changed, which can increase the potential to catalyze the degradation of negatively charged contaminants. In the work of Oliveros et al. (2021), the DCF degradation efficiency decreased with decreasing pH. Complete removal of DCF was achieved within 100 min at pH > 7, while at pH 6 and 5, the removal rates were 96.4% and 84.2%, respectively. This is slightly different for other AOPs, where, for example, an increase in pH causes a decrease in reaction efficiency, e.g., the UV/peroxymonosulfate (PMS)/Fe^2+^ process (Rehman et al., 2021). This can be explained mainly by the effect of the catalyst used and, at the same time, justifies the need to select the optimum catalyst depending on the reaction conditions for DCF removal. Mugunthan et al. (2019) investigated the effect of different pH values on the DCF degradation efficiency in a process catalyzed by ZnO-WO_3_ composite (zinc oxide doped with tungsten precursor). The visible radiation source was a 400-W halogen lamp. With an initial concentration of *C*_0[DCF]_ = 20 mg/l and a ZnO-WO_3_ dose = 800 mg/l, the highest removal of DCF (about 75%) was achieved at neutral pH = 6. This was due to the surface charge properties of ZnO-WO_3_ (pH_pzc_ = 7.35 ± 0.2), so the catalyst surface was positively charged, and diclofenac should be negatively charged.

The methylene blue removal in the advanced oxidation processes continues to attract considerable interest. In the Scholar database (Google Scholar Database, 2022), between 2019 and 2021, the total number of articles containing the phrase “advanced oxidation of methylene blue” was 67,700 (in 2019, 19,000; in 2020, 20,700; in 2021, 28,000).

In general, the efficiency of MB removal in photocatalytic processes is determined by the following parameters: initial concentration, pH of the solution, temperature, dose, and type of catalyst.

Before irradiation, the contaminants should be adsorbed on the surface of the photocatalyst to achieve adsorption–desorption equilibrium. Greater adsorption on the catalyst reaction site leads to increased MB degradation. Before MB photodegradation, the adsorption time (conducted in the dark) is usually from 30 min (El-Katori et al., 2020; Lee et al., 2019), but depending on the catalyst used, this time can be 60 min (Ziarati Saravani et al., 2019) or even 120 min (Nuengmatcha et al., 2019). In the photocatalytic process involving TiO_2_/NiFe_2_/reduced graphene oxide, approximately 55% MB adsorption was achieved after 60 min (Ziarati Saravani et al., 2019). In comparison, for pure TiO_2_, the adsorption efficiency was set to 38%. In contrast, by using a nanostructured Fe/FeS catalyst, Cabir et al. (2017) obtained about 18% adsorption of methylene blue after 30 min.

Most MB degradation work uses xenon, LED, halogen, mercury, and tungsten lamps ranging from a few W to 500 W. However, high-power lamps dominate (> 100 W) (Esmailli et al. 2018; Mahanthappa et al., 2019). Higher lamp power results in higher MB removal rates. For example, Nuengmatcha et al. (2019) studied the effect of visible light irradiation at different intensities (0 − 130 W). With an initial MB concentration *C*_0[MB]_ = 20 mg/l and a catalyst dose = 100 mg/l, a 25% removal rate was obtained for 40 W, 40% for 60 W, and 60% for 100 W. Increasing the irradiation intensity above 100 W had no significant effect on increasing the removal rate of DCF (62%). The increase in the removal rate of DCFs with increasing radiation intensity was due to an increase in the intensity of oxidative radical production, which was also confirmed by Liu et al. (2019).

The initial pH of the solution has a significant influence on the efficiency of MB photodegradation in visible light–driven processes, as it affects the interaction between the adsorbent (catalyst) and the adsorbate (MB). In a conventional process using pure titanium(IV) oxide, the value of pH_pzc_ is approx. 6.0–6.5. Kaur et al. (2018) achieved the highest degree of adsorption of MB on TiO_2_ at pH = 11 (from about 41% to about 82% depending on the sample), while at pH ≤ 6, the amount of adsorption was the lowest, ranging from about 1% to about 6%. The MB molecule is positively charged, so high pH (> pH_pzc_) favors adsorption on the catalyst surface as it is then negatively charged. Similar observations were noted by Esmaili et al. (2018). The authors investigated the effect of pH on the removal rate of MB using Fe/FeS nanopowder as the catalyst. The highest removal rate (96%) was obtained at pH = 11. Lowering the pH resulted in a severe efficiency drop to 78% at pH = 9 and 25% at pH = 4.

Typically, achieving a minimum removal rate of 90% required photocatalytic time ranging from 10 to 200 min, with an average time of 120–180 min. For example, Lee et al. (2019) needed 180 min to achieve a 97% removal rate of MB using a bimetallic Au/Pd nanocomposite catalyst supported by ZnO. In contrast, Selvaraj et al. (2019) required 90 min to achieve 93% dye removal. The shorter process time was probably due to the lower initial MB concentration (10 mg/l) and higher catalyst dose (360 mg/l). Increasing the photocatalyst dose may improve the MB removal efficiency (Mahanthappa et al., 2019). An increase in photodegradation efficiency was observed in the process using a CuS-CdS catalyst at concentrations ranging from 40 to 240 mg/l. The removal rate of MB ranged from 40% to nearly 100%, while the highest removal rate was found at a dose of 200 mg/l (nearly 100%). Higher catalyst doses probably cause aggregation of nanoparticles and their faster sedimentation. The so-called radiation shielding effect of excessive particles may also occur (Rauf & Ashraf, 2009). The photodegradation efficiency also decreases with increasing dye concentration. Bagherzadeh et al. (2018) investigated the effect of MB concentration on its photocatalytic degradation efficiency. An increase in DCF concentration from 10 to 20 mg/l resulted in decreased process efficiency from 92 to 73%. This phenomenon is characteristic of AOPs (Liu et al., 2018; Zotesso et al., 2017). With increasing concentration, the consumption of oxidative radicals is higher, and the probability of collision of oxidative radicals with dye molecules decreases (Zawadzki, 2021a).

### Visible Light Activation of Persulfate

**Fig. 4:**
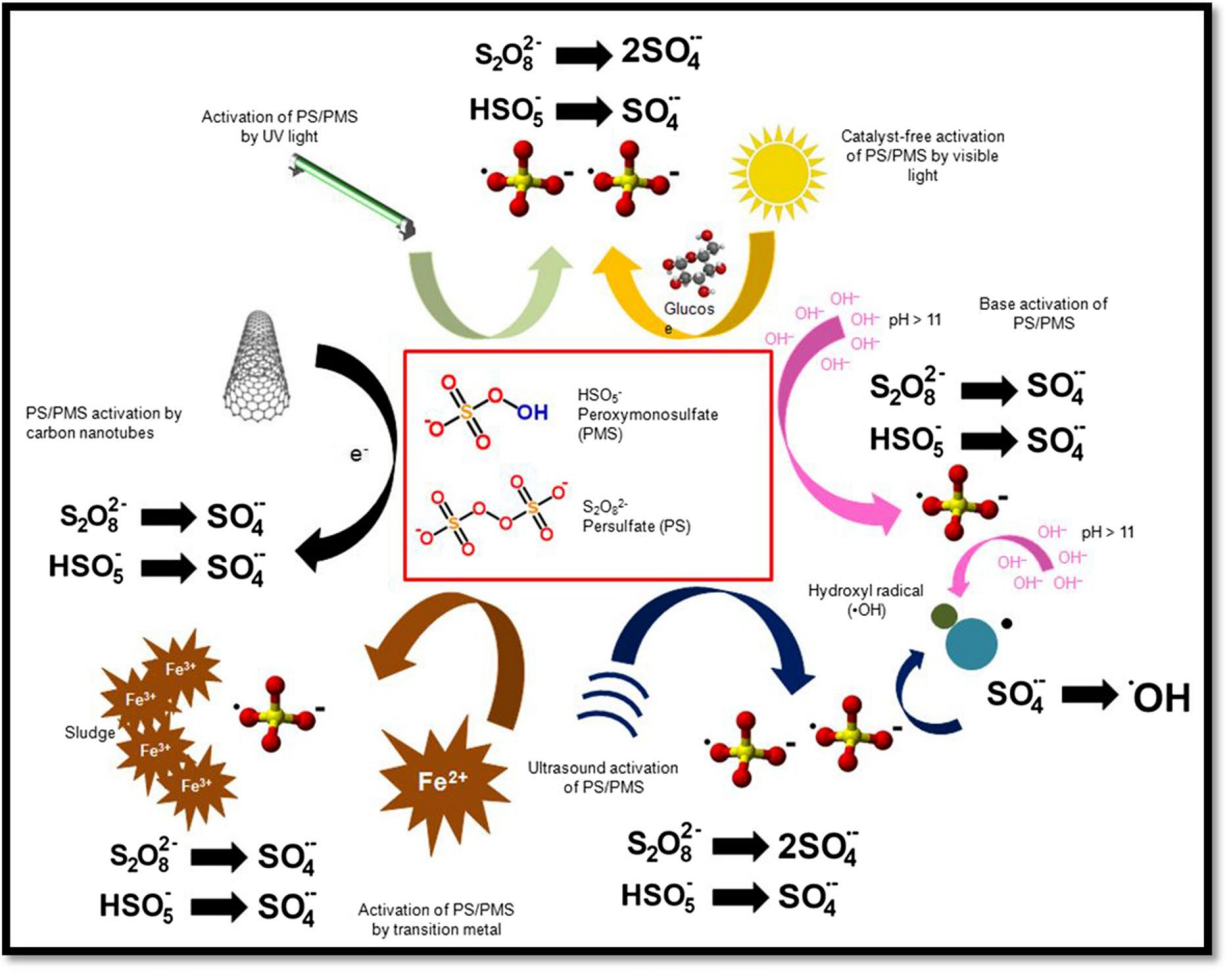
Selected methods of persulfate activation

In a sulfate radical oxidation process, a radical precursor (e.g., sodium persulfate ${\mathrm{Na}}_{2}{\mathrm{S}}_{2}{\mathrm{O}}_{8}$) requires activation. Persulfate without activation can only react with some organic compounds, and the efficiency of the process is significantly lower compared to that of activated persulfates. Without activation, the persulfate anion has an oxidizing potential about 33% lower than that of the sulfate radical (Karim et al., 2021; Zhu et al., 2019). While activation can be achieved by thermal, photolytic, sonolytic, and radiolytic actions (Criquet, Karpel & Leitner 2011; Chen & Su, 2012; Zhang et al., 2015; Ji et al., 2016; Ahmadi et al., 2019), the most used activation method is the application of low-oxidation transition metal ions such as Fe^2+^, Ni^2+^, Co^2+^, and Ag^+^. Current publications also include laboratory experiments on developing new activation methods for persulfate. These techniques include activation at high pH (> 11), electrolysis, the use of carbon nanotubes or polymers (polyimides), and ozone (Ding et al., 2020; Fernandes et al., 2021; Ren et al., 2019; Zou et al., 2021). Some of the selected persulfate activation methods are graphically shown in Fig. 4.

In persulfate (PS) activation processes, the energy transferred to the persulfate anion by UV light, ultrasound, or heat results in the cleavage of the peroxide bond and the formation of two sulfate radicals (Eq. (1)). The persulfate can also react with an electron donor from the transition metal to form a single sulfate radical (Eq. (2)) (Karim et al. 2020).

1 $${\mathrm{S}}_{2}{\mathrm{O}}_{8}^{2-}+\mathrm{energy input }\to 2{\mathrm{SO}}_{4}^{\bullet -}$$

2 $${\mathrm{S}}_{2}{\mathrm{O}}_{8}^{2-}+{\mathrm{e}}^{-} \to {\mathrm{SO}}_{4}^{2-}+{\mathrm{SO}}_{4}^{\bullet -}$$

An alternative method for visible light activation of persulfates has also received increasing attention in recent years. Alternative activation methods should be as effective and cost-efficient as conventional methods. Materials for visible light activation of persulfates can be acids (e.g., ascorbic acid) and sugars (e.g., glucose, sucrose) (Hou et al., 2020b; Watts et al., 2018; Zawadzki, 2019). Degradation of micropollutants in the presence of persulfate and visible light is also achieved in processes involving catalysts, e.g., TiO_2_ (Du et al., 2020), or combined methods, e.g., ultrasound and visible light–activated sodium persulfate (Zawadzki, 2021a). Recently, a promising approach to activate persulfates is the innovative material perylene diimide (PDI). PDI has excellent charge separation efficiency. Electron injection from PDI into PS can more efficiently produce active forms of oxidative radicals (Ji et al., 2021).

The generation of sulfate radicals was carried out in the presence of sodium persulfate (Na_2_S_2_O_8_), glucose, and visible light (an innovative activation method) in the research by Zawadzki (2021a). Glucose was essential to activate Na_2_S_2_O_8_ in visible light. Literature data indicate that the activation mechanism by glucose is similar to that by phenoxides (Ahmad et al., 2013; Watts et al., 2018). Glucose is an optically active substance (Ashenhurst 2022). An electron from glucose is transferred to persulfate and activates it; in turn, glucose is oxidized to products that can activate persulfate. Some functional groups, such as the carbonyl group, accept a negative charge, activating persulfate at near-neutral pH. Zawadzki (2021b) performed a study on the advanced oxidation of chlorfenvinphos from real treated municipal wastewater as stage IV of municipal wastewater treatment. Under optimal conditions (pH = 6; room temperature; Na_2_S_2_O_8_ dose = 20 mM; glucose dose = 100 mM; process time = 20 min), an 81% removal rate of CFVP was achieved. Irradiation of the solutions with visible light caused the glucose decomposition, electron transfer from sugar towards Na_2_S_2_O_8_ (activation), and oxidation of glucose to sodium persulfate activation products.

Besides the CFVP removal, studies show that diclofenac can be effectively removed during reactions in the presence of sulfate radicals generated in the presence of visible light. In AOPs, an important parameter is the pH of the solution, which affects the performance of oxidants and catalysts and the degradation degree of pollutants. Shao et al. (2017) investigated the effect of initial solution pH on DCF degradation during peroxymonosulfate activation by g-C_3_N_4_-modified Co_3_O_4_ nanoparticles (Co_3_O_4_-g-C_3_N_4_). It was observed that the first-order kinetic constant (*k*) decreases with increasing pH, which also affected the final removal rate of DCF. For example, in a strongly alkaline medium (pH = 11), a 75% removal rate of DCF was achieved. In contrast, in a strongly acidic environment (pH = 3), nearly 100% removal rate of DCF was achieved. As explained by Ao et al. (2018) and Xia et al. (2020), under pH < 7, the predominant radicals are ${\mathrm{SO}}_{4}^{\bullet -}$, whereas above pH > 7, sulfate radicals are converted to –OH radicals by reacting with ${\mathrm{O}}_{2}^{\bullet -}$. At pH = 11, Urán-Duque et al. (2021) observed a significant inhibition of the degradation process. Han et al. ([Bibr CR72][Bibr CR72]) also used BiFeO_3_ microsphere–activated (BFO) PMS to degrade diclofenac. Bismuth ferrite (BiFeO_3_, BFO) is a heterogeneous catalyst used in the work of Hussain et al. (2018) and Ouyang et al. (2020), among others, due to its multiferroic properties and high chemical stability, i.e., resistance to strong acids and bases. The authors of this study achieved an approximately 82% removal rate of DCF using a BFO dose of 500 mg/l, a PMS dose of 0.5 mM, and a processing time of 60 min. The source of Vis radiation was a led lamp. The operational parameters for the removal of diclofenac in the BFO/PMS process such as process time, pH, BFO dose, and PMS dose were also determined. Firstly, the efficiency of DCF elimination is affected by the reaction time, i.e., the longer the reaction time, the higher the removal degree (about 60% after 20 min of reaction and 80% after 60 min of reaction under the following conditions: DCF concentration = 0.025 mM, BiFeO_3_ dose = 300 mg/l, and PMS dose = 0.5 mM). The highest degree of DCF removal was obtained after 60 min. Afterwards, the pH value of which was indicated as optimal at pH = 3. However, iron leaching was observed at pH = 3, which did not occur at higher pH. At pH = 3, the highest degree of pharmaceutical removal was obtained (approx. 80%) under the following conditions: DCF concentration = 0.025 mM, BiFeO_3_ dose = 300 mg/l, and PMS dose = 0.5 mM. The reduction in sulfate radical generation may also have been due to an increase in the mutual repulsion between BFO and PMS. The authors observed a significant increase in efficiency between the BFO dose of 400 mg/l and 500 mg/l (from about 65% to about 82%), whereas an increase in dose to 600 mg/l resulted in virtually no increase in removal efficiency. Also for the PMS, generally an increase in dose resulted in an increase in DCF removal with the optimum value at 0.5 mM PMS.

Many studies on MB degradation by PS or PMS in the presence of visible light primarily focus on the activation of PS or PMS with solid catalysts (e.g., TiO_2_, ZnO, carbon nanotubes, or other modified photocatalysts) and then the role of visible light. However, there are few studies on the oxidation of MB by sulfate radicals without the introduction of solid catalysts (e.g., sugars, acids, and other electron sources). As presented in the literature (El-Sheshtawy et al., 2020; Habib et al., 2021; Sun et al., 2020), the degradation of methylene blue is determined by the following operational parameters; among others are as follows: initial MB concentration, process time, catalyst dose (PMS/PS), pH, lamp type, and power.

Zawadzki (2019) determined the operational parameters for MB removal in the visible light oxidation process with sodium persulfate (Na_2_S_2_O_8_) activated by glucose and sucrose, such as reaction time, pH, glucose/sucrose dose, and Na_2_S_2_O_8_ dose. The highest degree of MB degradation (84%) was observed in the presence of sodium persulfate (6.5 mM) after 90 min of visible light irradiation for the process carried out in the presence of glucose (100 mM) at pH = 12. It was determined that the radicals responsible for the decolorization of methylene blue were ${\mathrm{SO}}_{4}^{\bullet -}$, •OH, and ${\mathrm{O}}_{2}^{\bullet -}$. At pH = 12, hydroxyl radicals were mainly responsible for the degradation of methylene blue. The results were similar to those obtained by Watts et al. (2018).

For reactions carried out in the presence of sulfate radicals, xenon, LED and tungsten lamps are used, similar to photocatalytic processes. However, a significant difference in the lamp power used has been observed. Namely, lower-wattage lamps (up to 50 W) are used for MB removal processes in the presence of persulfates/peroxymonosulfates and catalysts, which may probably be due to the applied synergistic effect in these processes between catalysts and PMS/PS. The combination of photocatalysis and PMS activation promotes charge separation in the photocatalytic system as an electron capturing agent and improves light utilization in the photocatalyst (Hu et al., 2019).

The combination of photocatalysis and PMS activation also extends the pH range in which the process can still be carried out efficiently. For example, Tang (2020) obtained a BiVO_4_ catalyst to activate PMS. First, a 99% removal rate of MB was achieved after 90 min of reaction (conditions as in Table 2). Then, increasing the pH from 4 to 10, a similar degree of BM removal determined as 95–99% was obtained. At pH = 2, the reaction efficiency decreased slightly to about 78%. The presented method may therefore be suitable for the treatment of colored effluents characterized by a wide pH range, as the efficiency of dye decomposition in each of the pH ranges examined was higher than 75%.

In general, the removal rate in all analyzed processes depended on the reaction time. The optimal process time is also an important parameter from an economic point of view (reactor volume, electricity costs, automation, electronics). Typically, a period of 60–90 min is needed to remove MB concentrations from 2 to 40 mg/l. To remove 100% MB with a concentration of 3.2 mg/l, Sabri et al. (2020) needed 90 min (for the conditions set in Table 2). The time required for the complete removal of MB can be reduced by increasing the dose of PS or PMS. For example, in the study by Rizal et al. (2021), nearly 100% MB degradation was achieved after 70 min (PS concentration = 2 mM; Ag/Mn_3_O_4_/graphene catalyst = 500 mg/l). However, by increasing the dose to 4 mM, this time was reduced to 40 min, and at a dose of 12 mM to 30 min.

Han et al. (2020b) and Rizal et al. (2021) also investigated the effect of initial MB concentration on the dye removal rate. Both studies confirm that higher dye concentrations inhibit the radical reactions with dye molecules. Furthermore, a high concentration of molecules can lead to competition effects between dye molecules, reaction by-products, and generated radicals (Zawadzki, 2021a).

### Visible Light–Driven Photoelectrocatalysis

Photoelectrocatalysis (PEC) is a combined process of photocatalysis and electrochemistry (Hou et al., 2020a; Xu et al., 2019). PEC primarily aims to suppress the negative recombination phenomenon of hole-electron pairs generated by photocatalysis.

**Fig. 5:**
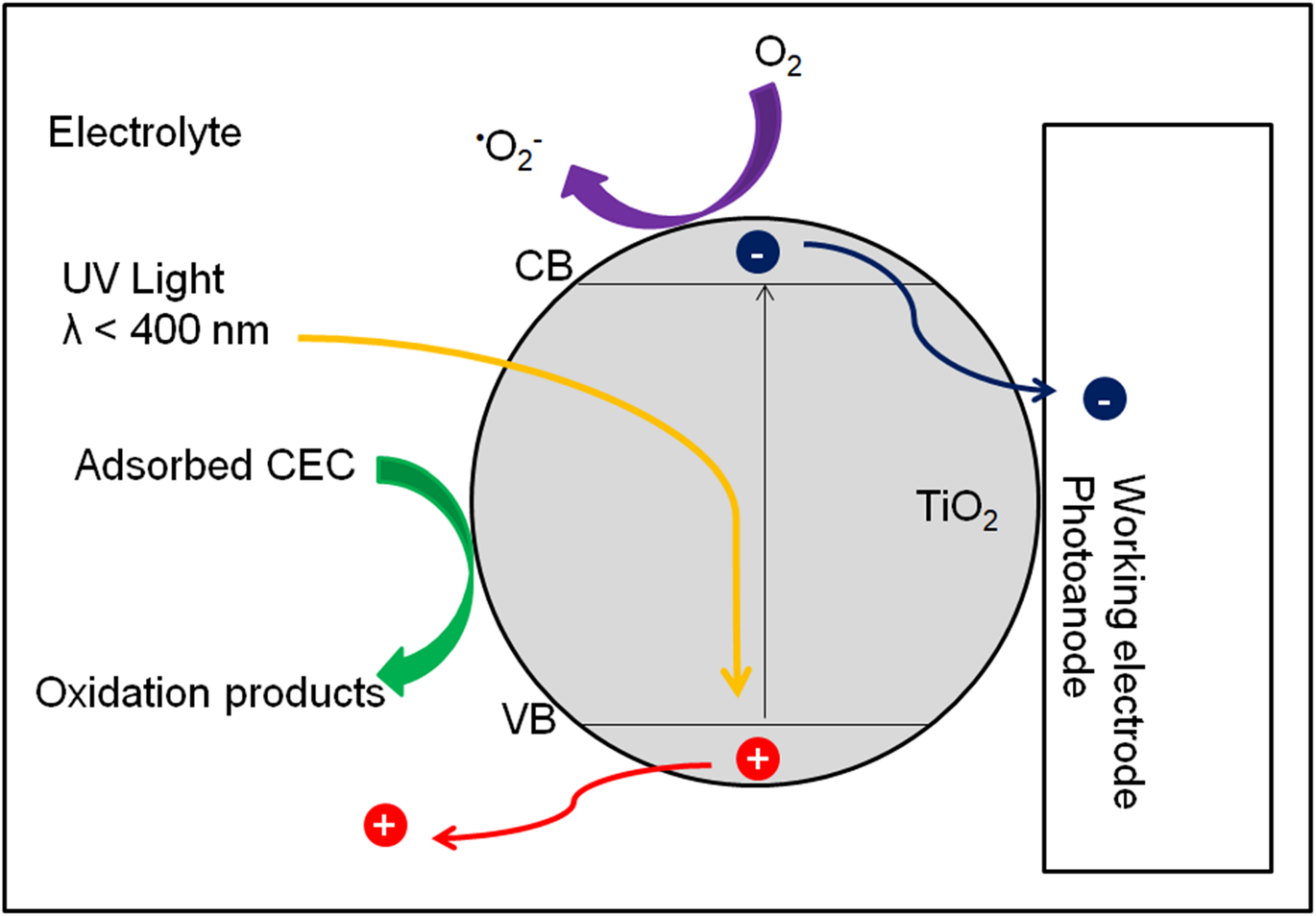
Mechanism of photoelectrocatalysis in the presence of *n*-type semiconductor (TiO_2_). Own study based on the literature (Bessegato, Guaraldo & Zanoni 2014; Bessegato et al., 2015; Ge et al., 2016)

In this method, a semiconductor is attached to the surface of a conductive substrate and used as a photoelectrode. Photogenerated holes on the surface of the semiconductor trigger oxidation reactions and electrons flow through the counter electrode where reduction reactions take place. Thus, charge recombination is minimized, and the quantum efficiency of the photocatalytic process is improved. Irradiation of an *n*-type semiconductor (e.g., TiO_2_) with radiation of an energy higher than the activation energy results in the generation of charge carriers. Most of the research on photocatalysis is devoted to the removal of contaminants from a liquid medium in which the semiconductor is held in suspension. This way of using catalytic nanoparticles introduces the need to separate them from the liquid phase after the photodegradation process. This can be avoided by coating the conductive substrate with film-forming particles (immobilization). In the PEC process, the dissolved organic substances in the electrolyte are oxidized through the holes formed, and the electrons are transported to the conductive substrate (Fig. 5) (Bessegato et al., 2015).

PEC shows an advantage over photocatalysis because it applies a potential to the photoanode on which the catalyst is deposited. This configuration allows for more efficient separation of the charges (e/h^−+^) formed in the process, thereby increasing the lifetime of electron–hole pairs (Hou et al., 2020a, 2020b; Li et al., 2018; Su et al., 2016). Photoelectrocatalysis has been shown to efficiently degrade chlorfenvinphos (Fernández-Domene et al., 2019; Roselló-Márquez et al., 2021), diclofenac (Cheng et al., 2015; Liu et al., 2017b), and methylene blue (Rosa et al., 2020; Wu et al., 2019). Advances in electrochemistry and materials science (new materials active in visible light) have led to increased interest in using photoelectrochemical processes to eliminate CECs. Photoelectrocatalysis and visible light–driven photocatalysis have emerged as promising strategies for clean, low-cost, and environmentally friendly renewable energy production and removal of contaminants (Pan et al., 2020; Zhong et al., 2020).

In the work of Fernández-Domene et al. (2019) and Roselló-Márquez et al. (2021), the photoelectrochemical decomposition of chlorfenvinphos with tungsten trioxide (WO_3_) nanotubes was investigated. Chlorfenvinphos solutions with an initial concentration of 20 ppm were treated with visible radiation, the source of which was a 1000-W xenon lamp. Innovative nanostructured electrodes produced by anodizing tungsten and annealed at 400 °C and 600 °C were added to the treated solutions. The process studied achieved a 95% removal rate of CFVP under the operating parameters shown in Table 2. A more than 65% reduction in total organic carbon was also achieved.

In a similar study, Roselló-Márquez et al. (2021) used a 500-W xenon lamp (visible light source). Under the room temperature and after 24 h of treatment, a 95% removal degree of CFVP was obtained. The analysis of reaction intermediates during photoelectrochemical oxidation of CFVP also showed interesting results. As reported by Farré et al. (2005), detoxification of chlorfenvinphos is achieved when the TOC remaining in solution is below 10 mg/l. Otherwise, increased toxicity may be due to generated by-products. Depending on the process (e.g., photo-Fenton, radiolytic decomposition), the oxidation by-products may differ. In the literature, CFVP degradation by-products are reported to be, for example, 2-hydroxy-1-(2,4-dichlorophenyl)vinyl diethyl phosphate, 2,4-dichlorobenzoic acid, dicarboxylic acid, 2,4-dichlorophenol, triethyl phosphate, and 4-hydroxybenzoic acid (Klamerth et al., 2009; Bojanowska-Czajka et al., 2010; Roselló-Márquez et al., 2021). The by-products are therefore aromatic acids or esters, but also toxic products, e.g., 2,4-dichlorobenzoic acid or triethyl phosphate. Photoelectrocatalysis in the presence of WO_3_ resulted in the generation of, among others, 2,4-dichlorobenzoic acid, triethyl phosphate, and ethyl dimethyl phosphate (Roselló-Márquez et al., 2021).

Similar efficiency was obtained during the removal of diclofenac in PEC. Liu et al. (2017b) used a photoelectrocatalytic purification system in the presence of PS. The photoelectrocatalytic system consisted of a c-Bi_2_MoO_6_ photoanode and a copper foil cathode. At an applied voltage of + 1.5 V and an initial solution pH value of 5.62, the removal efficiency of DCF with an initial concentration of 10 mg/l was 86.3% with the addition of 10 mM PS.

In visible light photoelectrocatalysis, voltages between + 0.4 and + 2.0 V are generally used for CFVP degradation. In general, the degradation degree increases with increasing voltage, whereas the increase is not relatively high. For example, a study by Sun et al. (2018) found that the degradation efficiency of DCFs increases with increasing polarization potential. In a photoelectrochemical process involving a composite obtained by combining bismuth vanadate (BiVO_4_) and graphitic carbon nitride (g-C_3_N_4_), a more than fourfold increase in DCF removal rate was obtained after 2 h of treatment when the polarization potential was increased from 0 to + 1 V. The increase in polarization potential to + 1.5 V increased the removal degree from 29.4 to 31.2%. Similar observations were also confirmed in a review paper by McMichael et al. (2021).

A significant increase in the efficiency of photoelectrocatalysis can be achieved by combining composite materials and other oxidants, such as hydrogen peroxide, as shown in the study of McMichael et al. (2021). The authors investigated the effect of hydrogen peroxide on the degradation efficiency of diclofenac in the presence of visible light. Ten millimolars of H_2_O_2_ was chosen as the optimal concentration, due to the increase in the removal rate of DCF to 62.3% after 180 min of reaction. A higher concentration of H_2_O_2_ (15 mM) resulted in a lower removal rate due to the probable reaction of excess H_2_O_2_ with the generated ^•^OH radicals, thus inhibiting the degradation process. Similar conclusions were postulated by Ku et al. (2005) and Ziembowicz et al. (2017).

The idea behind photoelectrocatalytic processes is to generate highly reactive oxidative radicals. It can be thought that, mainly, hydroxyl radicals (^•^OH) and, to a lesser extent, ^•^O_2_^−^, H_2_O_2_ and h^+^ radicals are responsible for the decomposition mechanism in visible light–driven photoelectrocatalysis, as shown in Cheng et al. (2015).

In the last few years (2017–2021), there have been few studies on the elimination of MB by photoelectrocatalytic processes under visible light. The Scopus database contains 29 publications for 2017–2021 containing the keywords “photoelectrocatalytic degradation of methylene blue under visible light,” with the majority (10 articles) published in 2017.

The degradation of MB in a photoelectrocatalytic process is determined by the following process parameters, among others: voltage, effective photoelectron area, pH, type of electrolyte, and its concentration.

Light-sensitive modified catalysts (e.g., CdS, TiO_2_, ZnO, WO_3_, BiVO_4_) have been used for the degradation of MB in visible light photoelectrocatalytic processes. For example, Liu et al. (2017a) modified TiO_2_ with NH_4_F (source of F), yielding a visible light–active material; in the presence of which, the MB removal rate was 92% compared to pure TiO_2_ at 50%. The absorption band towards visible light was shifted by also using F-doped tin oxide (FTO) and WO_3_/BiVO_4_ (Thongthep et al., 2021).

Sampath et al. (2016) investigated the photoelectrocatalytic activity of the ZnO/porous silicon (PS) over the applied voltage from − 6 to + 6 V. The highest photoelectrocatalytic activity (c.a. 96% of MB removal at an initial concentration of 20 mg/l after 105 min) was obtained for a negative voltage (− 6 V). This was explained, among others, by the efficient separation of charge carriers by driving the photogenerated holes through an external circuit to the counter electrode during negative voltage. Increasing the voltage to 0 V systematically decreased the degradation efficiency (up to 85% at 0 V), while further increasing the voltage increased the MB removal efficiency (up to 88% at + 6 V). Different results were presented by Zhao et al. (2019), using an indium oxide (In_2_O_3_)-doped ZnO catalyst for MB removal (*C*_0[MB]_ = 20 mg/l, process time = 60 min). The photodegradation efficiency depended, among others, on the amount of In_2_O_3_ in ZnO and the applied voltage. For the optimal In:Zn ratio of 0.05:1 (photocurrent density = 264 µA/cm^2^) and the applied voltage of + 0.2 V, a 95% removal rate of MB was achieved. At a lower voltage (+ 0.1 V), an efficiency of 86% was achieved, and at the highest voltage tested (+ 0.4 V), the lowest MB removal rate of 79% was observed. In general, positive voltages ranging from + 0.2 to + 6 V are used in studies on MB removal in photoelectrocatalytic processes in the presence of visible light.

The authors of most works use photoelectrodes with an effective area between 0.0071 and 50 cm^2^ (Gandamalla et al., 2021; Liu et al., 2017a; Nareejun & Ponchio, 2020). Larger photoelectrode areas can drastically reduce the process efficiency due to the possible introduction of more defects in the photoanode (cathode) materials (Li & Li, 2017).

In a study by Liu et al. (2017a), the effect of solution pH on the removal rate of MB with a concentration of 10 mg/l was tested (for conditions given in Table 2). An increase in the degradation rate of MB was observed from about 82% (at pH = 3.14) to about 98% (at pH = 9.94). It was shown that the MB degradation reaction occurred more efficiently at high pH due to a change in the isoelectric point (pH_pzc_) value of the F-TiO_2_ catalyst. The pH_pzc_ of the F-TiO_2_ catalyst (F concentration = 15 wt%) was determined to be 6.72. Therefore, the catalyst surface at pH > 6.72 was negatively charged, which favored the adsorption and photodegradation of the positively charged MB molecule.

Gandamalla et al. (2021) performed an interesting study on the effect of temperature on the photoelectrocatalytic decomposition of methylene blue. Namely, an increasing temperature increased the removal rate of MB. For example, at 30 °C, the dye removal rate was 97.3%, while at 50 °C, the degradation rate was 99.09%. The authors attributed the increase in photodegradation efficiency to increased collisions between molecules at higher temperatures and more MB molecules adsorbed on the catalyst surface.

## Overview of Visible Light–Driven AOP Mechanism and Degradation

AOPs typically have complex reaction mechanisms, and more than 150 steps have been developed to describe them (Stanbury, 2020). It is also believed that the chemical mechanisms of oxidation in these systems involve multiple radical reactions (Wang et al., 2020; Ghime and Gosh 2020). The general mechanism of the photocatalysis process includes the following processes (Eqs. (3) − (5)):

3 $${\mathrm{TiO}}_{2}\stackrel{\mathrm{hv}}{\to }{\mathrm{e}}^{-}+{\mathrm{h}}^{+}$$

4 $$\mathrm{Ti}\left(\mathrm{IV}\right)-{\mathrm{OH}}^{-}+{\mathrm{h}}^{+}\leftrightarrow \mathrm{Ti}\left(\mathrm{IV}\right)-{}^{\bullet }\mathrm{OH}$$

5 $$\mathrm{Ti}\left(\mathrm{IV}\right)-{\mathrm{OH}}_{2}+{\mathrm{e}}^{-}\leftrightarrow \mathrm{Ti}\left(\mathrm{IV}\right)+{\mathrm{OH}}^{-}+{\mathrm{H}}^{+}$$

When the catalyst absorbs the radiation, active transition complexes are generated on the surface of the semiconductor, resulting in the generation of ^•^OH radicals, which strongly oxidize organic chemicals. The photogenerated electrons can react with H_2_ and O_2_ dissolved in water to form H_2_O_2_, which can be photodecomposed into ^•^OH radicals (Girón-Navarro et al., 2021). For the practical application of photocatalytic processes, it is important to increase the efficiency of the photocatalysis process in visible light, eliminate the agglomeration of semiconductor particles, reduce the phenomenon of blocking active sites, and increase the efficiency of separation of catalyst particles from the reaction mixture after the treatment process. Therefore, numerous semiconductor modifications are currently used to facilitate the absorption of visible light and simultaneously overcome the difficulties occurring in conventional photocatalysis (Pirhashemi et al., 2018; Wangab et al., 2016; Zawadzki et al., 2021).

As previously mentioned, photoelectrocatalysis combines photocatalytic and electrochemical oxidation processes. When light photons (hv) with an energy higher than the activation energy (*E*_g_) reach the surface of a semiconductor (S), which is deposited on a solid surface, charge carriers are generated (Eqs. (6)–(8)). The recombination of photogenerated electrons and holes occurring in the photocatalytic process is retarded by an applied bias potential (Alulema-Pullupaxi et al., 2021; Peleyeju & Arotiba, 2018).

6 $$\mathrm{S}+\mathrm{hv}\to {\mathrm{e}}_{\mathrm{CB}}^{-}+{\mathrm{h}}_{\mathrm{VB}}^{+}$$

7 $${\mathrm{h}}_{\mathrm{VB}}^{+}+{\mathrm{H}}_{2}\mathrm{O}\to {}^{\bullet }\mathrm{OH}+{\mathrm{H}}^{+}+{\mathrm{e}}^{-}$$

8 $${\mathrm{h}}_{\mathrm{VB}}^{+}+{\mathrm{OH}}^{-}\to {}^{\bullet }\mathrm{OH}$$

As shown in Eqs. (1) and (2), sulfate radicals are generated by activation of the ${\mathrm{SO}}_{4}^{\bullet -}$ precursor (peroxydisulfate (PDS)) through energy transfer to the persulfate anion or reaction with an electron donor from the transition metal. In general, the essence of generating the ${\mathrm{SO}}_{4}^{\bullet -}$ radical is to break the O–O bond in PDS. The O–O bond distance in PDS is 1.497 Å and must be severed in order to generate the sulfate radical (Ghanbari & Moradi, 2017). Instead of using energy-consuming UV lamps or transition metal ions that generate additional costs, materials for PDS activation under visible light, such as organic promoters, are currently being developed (Hu et al., 2021; Zawadzki, 2022).

In general, in visible light–driven AOPs, the degradation mechanism of emerging contaminants is similar to the conventional process. An overview of the visible light–driven AOP degradation mechanism of selected target pollutants is presented in Table 3. Free radicals, such as hydroxyl or sulfate radicals, are responsible for degradation of pollutants. ${\mathrm{O}}_{2}^{\bullet -}$, h^+^, and OH^−^ radicals are also involved in photodegradation. Depending on the structure of the compound, degradation may involve a number of intermediate reactions, for example dechlorination, decarboxylation, C–N bond cleavage, and hydroxylation reaction. Finally, cleavage of the aromatic ring takes place. In dye degradation, *N*-deethylation, chromophore cleavage, and ring opening can take place, leading to a series of oxidation products with smaller molecular sizes (Diao et al., 2017; Lops et al., 2019). The degradation of azo bonds has been suggested as a possible mechanism for MB decolorization (Mahdavianpour et al., 2020).

**Table 3:** Degradation mechanism of selected target pollutants

Target pollutant	Process	A brief description of the degradation mechanism	References
Methylene blue	Peroxymonosulfate (PMS) activated by surface-tailored carbon quantum dots (CQDs)	In the presence of visible light, in the CQD/PMS system, the primary reactive species for MB oxidation are ${\mathrm{O}}_{2}^{\bullet -}$ and h^+^ generated during PMS activation and excited CQDs. Under visible light irradiation, photogenerated electrons can activate PMS to generate highly reactive ${\mathrm{O}}_{2}^{\bullet -}$. CQDs can be excited by Vis radiation, resulting in the simultaneous generation of holes and electrons. The PMS molecule decays into a ${\mathrm{HO}}_{2}^{\bullet }$ radical and a sulfite ion. The ${\mathrm{HO}}_{2}^{\bullet }$ radical breaks into a proton and superoxide ion, causing MB to degrade. The presence of MB causes the absorption of oxidation holes and limits the recombination of hole-electron pairs and further promotes the photocatalytic activation of PMS. Alkaline environment may promote the generation of more reactive species in the CQD/PMS system	Han et al. (2020a)
Methylene blue	Degradation by sodium persulfate activated by glucose (PS/G/Vis)	Degradation mechanism caused by sulfate and hydroxyl radicals Glucose and sucrose are optically active substances; i.e., they tend to rotate the light plane and are active in visible light. The activation mechanism of persulfate may be due to the generation of Krebs cycle compounds during sucrose hydrolysis. When glucose is used, persulfate activation may result from the probable electron transfer from sugar towards PS. Higher degradation efficiency is observed while sucrose is used because sucrose is hydrolyzed into glucose	Zawadzki (2019)
Methylene blue	Persulfate oxidation in the presence of photoexcited dye	The main mechanism is the radical reaction caused by the reduction of PS by photogenerated electrons of the dye; the second mechanism is a non-radical reaction involving the transfer of electrons via the dye from the pollutant to the oxidized dye	Cai et al. (2019)
Methylene blue	Graphene-decorated titanium dioxide (TiO_2_) powders	Graphene in the composite (TiO_2_/graphene) can reduce the transfer between photogenerated electrons formed when visible light reaches the surface of graphene and TiO_2_. The photocatalytic activity of graphene-modified TiO_2_ is much higher than that of pure TiO_2_, confirming that there is a synergistic effect of graphene and TiO_2_. Crystallite growth due to nucleation and growth of seed crystals were observed which may contribute to the above effect. The mechanism of MB degradation may be due to the absorption of visible light by the graphene-TiO_2_ composite and to the generation of excited photoelectrons at the Fermi level, which will tunnel into the conduction band of TiO_2_ to overcome the Schottky barrier formed by the contact between graphene and TiO_2_. The presence of these injected electrons will then interact with the dye to start its degradation	Acosta-Esparza et al. (2020)
Chlorfenvinphos	Photocatalysis in the presence of pyruvic acid (PA)-doped TiO_2_ (TiO_2_/PA)	The photodegradation is mainly due to ${\mathrm{O}}_{2}^{\bullet -}$ radicals, then h^+^ and least OH^−^. In the model solution at pH = 3, due to the change in hydrophobic properties of TiO_2_ modified with organic acids, the mechanism involves the adsorption of CVFP on the catalyst surface, followed by cleavage of the aromatic ring by oxidizing radicals, mainly ${\mathrm{O}}_{2}^{\bullet -}$	Zawadzki (2020)
Chlorfenvinphos	Visible light–driven photoelectrochemical degradation in the presence of WO_3_ nanorods	Degradation in the presence of WO_3_ nanotubes occurs by cleavage of the aromatic ring (π-π*). The time evolution of the UV absorption spectra of CFVP took values greater than 0, which means that the degradation of CFVP probably takes place by opening the aromatic ring and then generating intermediate compounds Degradation by hydroxyl radicals or directly with photodegenerated holes on the WO_3_ surface in semiconductor/electrolyte solution Further analytical work is needed to propose the full mechanism of chlorfenvinphos degradation in the presence of WO_3_ nanotubes	Fernández-Domene et al. (2019) Roselló-Márquez et al. (2019)
Chlorfenvinphos	Photodegradation by using WO_3_ nanostructures	^•^OH radicals are used as the main oxidizing agent for the degradation of CFVP. The photodegradation pathway of CFVP involves decomposition to a phosphate group, opening of the aromatic ring, or decomposition of the CFVP molecule by binding to phosphorus, with the formation of compounds without chlorine atoms and with longer aliphatic chains. The charge transfer mechanism for photogenerated holes in WO_3_ nanostructures occurs through the valence band	Roselló-Márquez et al. (2021)
Diclofenac	Photocatalysis in the presence of tungsten trioxide–doped TiO_2_ (TiO_2_-WO_3_)	The TiO_2_-WO_3_ catalyst has a higher photodegradation efficiency compared to pure TiO_2_, which confirms that the presence of WO_3_ increases the degradation efficiency of TiO_2_ in the modified catalysts, since the addition of WO_3_ decreases the value of the bandgap of the catalyst. The DCF degradation pathway mainly proceeds through dechlorination, decarboxylation, C‒N cleavage, and hydroxylation reaction. The DCF photodegradation step involves ring opening of aromatic compounds, which are then mineralized	Mugunthan et al. (2019)
Diclofenac	Photocatalysis in the presence of CQD-modified BiOCOOH photocatalysts (CQDs/BiOCOOH)	The CQDs greatly improved the visible light absorption by BiOCOOH, as well as interfacial charge transfer and separation. The CQDs/BiOCOOH contained new groups, such as CeN, NeH, and CeN/CeO, which enhanced the electron transfer ability of the material. The removal mechanism of DCFs in the presence of CQDs/BiOCOOH was not mainly related to adsorption due to the low surface area of the samples. It was found that ^•^OH, ${\mathrm{O}}_{2}^{\bullet -}$, h^+^, and e^−^ radicals were mainly involved in the degradation of DCFs during the treatment process under visible light. The main degradation pathway was via e^−^ reduction and ${\mathrm{O}}_{2}^{\bullet -}$ and ^•^OH addition reactions. The ${\mathrm{O}}_{2}^{\bullet -}$ radical was the most important radical in the photocatalytic degradation mechanism of DCF	Chen et al. (2018)
Diclofenac	Degradation by PMS activated by BiFeO_3_ microspheres (BFO)	The mechanism of PMS activation by BFO involves a series of reactions where a complex is formed between Fe^3+^ and ${\mathrm{HSO}}_{5}^{-}$; Fe^3+^ represents a site on the BFO surface; electron transfer from ${\mathrm{HSO}}_{5}^{-}$ to Fe^3+^ and the formation of a triple-bond Fe^2+^ and the ${\mathrm{SO}}_{5}^{\bullet -}$ radical; then, the reactions that occur lead to the formation of the ${\mathrm{SO}}_{4}^{\bullet -}$ radical. The ${\mathrm{SO}}_{4}^{\bullet -}$ radical can also react with H_2_O or OH^−^ to form the ^•^OH radical. Thus, DCF is degraded by both sulfate and hydroxyl radicals. Visible light irradiation provides an additional way of producing the hydroxyl and sulfate radicals and supports the Fe(III)-Fe(II)-Fe(III) redox cycle. Degradation of DCF may occur by decarboxylation, methyl oxidation, hydroxylation, benzene ring cleavage, C‒N bond cleavage, and dechlorination (cleavage of the C‒Cl bond by the ${\mathrm{SO}}_{4}^{\bullet -}$)	Han et al. (2020b)

## Conclusions

The wide range of contaminants entering surface waters with wastewater makes the application of conventional wastewater treatment technologies insufficient. Among the compounds found in water streams, there are micropollutants and substances of both natural (products of the metabolism of organisms) and anthropogenic origin can be found. In the second group, there are mainly compounds such as pharmaceuticals, pesticides, dyes, disinfection by-products (DBP), and polycyclic aromatic hydrocarbons (PAHs). Micropollutants belong to a group of chemical substances posing a particular risk to human health and life. They are the cause of the following, inter alia: cancer, mutations, poisoning, endocrine system disorders, defects in fetal development, and damage or death of embryos. These compounds are present in the environment in concentrations ranging from ng/l to µg/l.

Analysis of research in recent years has shown an increased interest in modifications of advanced oxidation processes, including those driven by visible light. As presented in this work, advanced oxidation processes driven by visible light have great potential to remove organic contaminants from water and wastewater, including diclofenac, chlorfenvinphos, and methylene blue. Undoubtedly, research into this purification technique has made considerable progress in recent years. Visible light–active catalysts, stable over a wide pH range and capable of simultaneous degradation of organic matter and, for example, hydrogen production, have been developed. Methods for the activation of persulfates with and without catalysts have been developed, and the visible light activity of persulfates in the presence of certain materials has been documented. A combination of AOPs, e.g., photocatalysis and electrochemistry, has also been developed to immobilize the catalyst on a solid substrate and use it as an electrode, and to reduce the negative recombination phenomenon of hole-electron pairs generated in the photocatalysis.

The following specific research recommendations are suggested for the next few years:

- Further study on the degradation of chlorfenvinphos from the water and wastewater (the lowest number of studies among the three CECs analyzed).
- More research into the influence of effective electrode surface area should be performed.
- More exploration of catalyst modifications to minimize defects in photoanode (cathode) materials and reduce energy consumption must be performed.
- Focus on the development of materials that ensure high stability and durability of catalysts and photoelectrodes.
- Achieving materials capable of activating persulfates or peroxymonosulfates without the use of a catalyst (catalyst-free persulfate activation).
- Perform more studies on the removal of mixtures of dyes, pesticides, and pharmaceutical substances.
- Enhance the toxicological study of advanced oxidized solutions (e.g., MicroTox® analysis *Aliivibrio fischeri* bacteria or toxicity analyses with aquatic plant, e.g., *Lemna minor*).

## Data Availability

The datasets used and/or analyzed during the current study are available from the corresponding author on reasonable request.

## Abbreviations

AOPs: Advanced oxidation processes
BCF: Bioconcentration factor
*C*_0[CFVP]_: Initial concentration of chlorfenvinphos
*C*_0[DCF]_: Initial concentration of diclofenac
*C*_0[MB]_: Initial concentration of methylene blue
CECs: Contaminants of emerging concern
CFVP: Chlorfenvinphos
DCF: Diclofenac
*E*^0^: Oxidation potential
EC_50_: Median effective concentration
*E*_g_: Activation energy
$${\mathrm{HSO}}_{5}^{-}$$: Peroxymonosulfate
*K*_OC_: Organic carbon–water partition coefficient
*K*_OW_: *n*-Octanol/water partition coefficient
LC_50_: Median lethal concentration
LED: Light-emitting diode
MB: Methylene blue
MPs: Microplastics
MSAs: Methanesulfonic acids
Na_2_S_2_O_8_: Sodium persulfate
NSAID: Non-steroidal anti-inflammatory drug
^•^OH: Hydroxyl radical
PDS: Peroxydisulfate
PEC: Photoelectrocatalysis
pH_pzc_: PH of the point of zero charge
PMS: Peroxymonosulfate
PS: Persulfate
${\mathrm{S}}_{2}{\mathrm{O}}_{8}^{2-}$: Peroxydisulfate/persulfate ion
$${\mathrm{SO}}_{4}^{\bullet -}$$: Sulfate radical
TBBPA: Tetrabromobisphenol A
UV: Ultraviolet
Vis: Visible
